# The effects of processing and sequence organization on the timing of turn taking: a corpus study

**DOI:** 10.3389/fpsyg.2015.00509

**Published:** 2015-05-13

**Authors:** Seán G. Roberts, Francisco Torreira, Stephen C. Levinson

**Affiliations:** Language and Cognition Department, Max Planck Institute for PsycholinguisticsNijmegen, Netherlands

**Keywords:** turn-taking, processing, sequence organization, frequency, concreteness, surprisal, random forests

## Abstract

The timing of turn taking in conversation is extremely rapid given the cognitive demands on speakers to comprehend, plan and execute turns in real time. Findings from psycholinguistics predict that the timing of turn taking is influenced by demands on processing, such as word frequency or syntactic complexity. An alternative view comes from the field of conversation analysis, which predicts that the rules of turn-taking and sequence organization may dictate the variation in gap durations (e.g., the functional role of each turn in communication). In this paper, we estimate the role of these two different kinds of factors in determining the speed of turn-taking in conversation. We use the Switchboard corpus of English telephone conversation, already richly annotated for syntactic structure speech act sequences, and segmental alignment. To this we add further information including Floor Transfer Offset (the amount of time between the end of one turn and the beginning of the next), word frequency, concreteness, and surprisal values. We then apply a novel statistical framework (“random forests”) to show that these two dimensions are interwoven together with indexical properties of the speakers as explanatory factors determining the speed of response. We conclude that an explanation of the of the timing of turn taking will require insights from both processing and sequence organization.

## 1. Introduction

Imagine a species that squawks at conspecifics. If it only has one message type (signaling e.g., “Here I am”), messages will have low information value. If there is only one rule of use, namely “one at a time,” communication will exhibit turn-taking, but not much other sequential patterning. Marmoset communication perhaps come close to this (Takahashi et al., 2013). Human communication differs radically on both dimensions: there is immense complexity on the informational parameter as well as the sequential one (Levinson, 2013b). In this paper we explore how these two parameters conspire to explain the temporal properties of human communication.

The core ecological niche for language use is in conversation: that is where language is learnt and the bulk of it is used. A key property of conversation is that participants take turns at talking. This is a demanding environment for language comprehension and production: So short is the average transition between turns that participants in a conversation must often simultaneously comprehend the current turn and plan the next turn (Levinson, 2013a). This suggests that demands on processing such as low frequency words or turns with dense information (Piantadosi et al., 2011) or more abstract concepts (Walker and Hulme, 1999) should influence the timing of turn transitions. That is, the duration of gaps between turns may reflect the amount of processing required to comprehend the previous turn and plan the upcoming turn.

Equally, however, conversational language use is characterized by two striking constraints. The first is a turn-taking system which minimizes gaps and discourages overlaps (Sacks et al., 1974); this is at least partially normative (interrupting is after all rude). The second is the mapping of structure across turns: a greeting is responded to with a greeting, a question (preferably) by an answer, an offer by an acceptance or declination, and so forth (Schegloff, 2007). This suggests that the major constraints come from interaction in context, and that the timing of turn-taking is above all sensitive to the constraints of sequence organization (Sacks et al., 1974; Schegloff, 2007). Studies from the field of conversation analysis demonstrate that the timing of turn taking may be sensitive to these constraints. Long gaps (i.e., of more than 700 ms) between turns are generally avoided in part because participants may be competing to take a turn at talk and it is the first speaker who takes the floor that generally keeps it. But in addition delayed turn transitions are interactionally marked in some interactional sequences, especially those in which an initial turn sets up an expectation for a specific type of response, as in questions and answers, offers and their uptake, requests and their compliance, etc. (see Stivers et al., 2009; Kendrick and Torreira, 2015). Hence a long pause after a request can be read as presaging non-compliance (Levinson, 1983). All of this suggests that interactional constraints could be of equal or greater importance for the timing of turn taking than simple processing constraints. Likewise, by the rules of turn-taking, certain types of utterances such as backchannels and repairs do not appear to be subject to the usual turn-taking constraints (i.e., avoidance of overlaps and long gaps) and may appear in overlap or be overlapped more frequently than other types of utterances (see Levinson et al., 2015). In sum, then, turn timing is sensitive to the normative structure of turn-taking and the sequential structure of conversation. Participants do not seem to begin a turn as soon as they have sufficiently processed the prior turn and planned their own turn, but rather hold off speaking until the other has finished their turn. For example, speakers generally identify possible points where a turn transition would be relevant in the interlocutor's turn before launching articulation of their own turn (see Levinson et al., 2015; Torreira et al., 2015; Bögels and Torreira, in press). On the other hand, speakers may begin a turn at talk without having fully planned their turn, by using filled pauses (e.g., “uh,” “um”) at the beginning of their turn in order to “buffer” their comprehension or planning (Clark and Fox Tree, 2002).

At the same time, it is unlikely that there is no relationship between the duration of turn transitions and cognitive processing requirements. It may simply not be possible to plan and launch an interactionally relevant turn following an extremely long, syntactically torturous sentence spoken extremely quickly. Teasing these two domains apart is not easy. Regarding the processing constraints, effects may be small and measures of such information may be difficult to compute. Real conversations, unlike controlled psycholinguistic experiments, are also subject to a large amount of noise. The ideal dataset would include a wide range of utterance types, but natural conversation is inherently subject to skewed distributions. This means that measures such as the frequency of words in a turn and the length of a turn will often be correlated. In order to get a reasonable sample, a large database of automatically processable conversation is needed. Such a quantitative approach goes rather against the tradition of work in conversation analysis, which is largely qualitative in nature, focusing on specific phenomena observed in close detail. However, in recent conversation analytic work, quantitative measures have increasingly been applied to qualitative coding (e.g., Clayman et al., 2007; Stivers et al., 2009). For example, interesting insights on the time course of language planning during turn-taking can be provided by controlling the sequential interactional context and other contextual relevant variables (e.g., several corpus studies on the timing of turn transitions in question-answer sequences, Stivers et al., 2009; Stivers and Enfield, 2010; Strömbergsson et al., 2013; Torreira et al., 2015). This demonstrates that, while qualitative analysis is often a powerful tool for explaining conversational phenomena, it is also possible to uncover and interpret systematic trends in a quantitative dataset provided that the researcher exerts some degree of control over the relevant contextual factors.

The Switchboard corpus (Godfrey et al., 1992; Calhoun et al., 2010) strikes a reasonable balance between the requirements of the two approaches, from theories of processing difficulty on the one hand, and the careful coding of conversational instances on the other. Tens of thousands of turns have been automatically collected and segmented, as well as hand-transcribed for a range of dialogue acts (e.g., different types of questions, statements, backchannels) relevant to sequence organization (see below). The aim of this paper is to assess to what extent measures of sequential organization on the one hand and cognitive processing on the other can explain the timing of turn taking. We use the statistical framework of Random Forests, explained below, to compare the importance of different variables in the distribution of transition times between turns.

This paper asks the following basic question: does sequence organization matter for the timing of turn taking beyond a battery of processing variables known to affect language processing? More precisely, do measures of sequence organization, albeit the coarse measures that are possible to extract from large corpora, contribute to the explanation of the timing of turn taking over and above measures of cognitive processing?

The amount of data and the number of variables makes the number of individual queries that can be asked of this kind of data very large. Also, as this paper shows, many variables are correlated, making it difficult to assess the strength of a relationship in isolation. By answering the question above and getting a “big picture” impression of the data, we hope to provide a map to fruitful future research.

The next section introduces the phenomenon of turn taking in interaction. Next, some predictions are made regarding how various cognitive processing and sequence organization measures should be related to the timing of turn taking. A short introduction to random forests is given before presenting the methods and results.

## 2. Turn taking in interaction

Conversations take place between two or more speakers who typically take turns at talk, usually minimizing overlapping talk (“overlaps”) and long turn transitions without talk (“gaps”). The “floor transfer offset” (FTO) provides a way of measuring gaps and overlaps in one single continuous variable (De Ruiter et al., 2006; Stivers et al., 2009; Heldner and Edlund, 2010). FTO is measured as the duration between the end of one turn and the beginning of another turn for pairs of turns involved in a floor transfer. FTO is negative if the turns overlap and positive if there is a gap between them. Cross-culturally, FTOs appear to be strikingly similar, with mean values ranging from 7 to 468 ms in a diverse sample of 10 languages (Stivers et al., 2009) (this range is small considering that the latency in the planning of a single word is of the order of 600 ms, see Levelt et al., 1999).

This paper focuses on conversations between two speakers. Throughout the paper, we will refer to “T1” as the turn prior to a floor transfer and “T2” as the turn following the floor transfer. We will refer to speaker A as the speaker of T1 and speaker B as the speaker of T2. Note that, in many cases, T2 becomes T1 for the next floor transfer in the conversation. Because of this, not all floor transfers involve the same kind of interactional contingency (e.g., a question and its answer vs. an answer to a question and an unrelated statement opening a new conversational sequence).

There are some previous studies of the distribution of FTOs. For example, Strömbergsson et al. (2013) find that FTOs for question-answer sequences are affected by the type of question asked, the type of response given and the topic of conversation. For example, responses were slower to open questions than wh-questions or polar questions. However, this study did not consider processing factors, analyzed the effects of T1 and T2 independently and was based on linear relationships within a restricted sequence type. Our study uses an order of magnitude more data, a wider range of sequence types and considers properties of both T1 and T2 together.

## 3. Cognitive planning and comprehension

Here we list some measures relevant to either production, comprehension, or both, whose importance we can readily check in the data to hand. We consider a number of hypotheses about how these might play a role in response times, measured in FTO.

### 3.1. Turn length

By definition, longer turns can have longer periods of overlap with another turn. Moreover, longer utterances are likely to be more complex than shorter utterances, requiring more processing. However, a longer utterance also gives more time for a listener to begin planning her own turn. Therefore, the predictions for effect of the length of T1 on FTO values are not clear without taking other measures of the content of the turn into account. On the other hand, the prediction for T2 length may be clearer. Planning a long utterance should generally take longer than planning a short one, so the FTO is expected to increase as the length of T2 increases.

### 3.2. Frequency

Psycholinguistic research has shown that word frequency plays a crucial role in ease of processing, both in comprehension and production. In lexical decision experiments for example (i.e., where participants must decide whether a displayed word is a real word or not, in as short a time as possible), frequent words are responded to more rapidly than infrequent words (Balota et al., 2007). This predicts that turns consisting of higher frequency words should be comprehended and produced faster, therefore reducing the turn transitions in which they are involved.

### 3.3. Concreteness

Words that refer to concrete entities (e.g., “ball”) contrast with words that refer to abstract entities (e.g., “justice”). Concreteness ratings have been shown to correlate with lexical decision times, with concrete words being comprehended faster (Schwanenflugel et al., 1988). Concrete words are also more easily recalled and produced than abstract words (Hanley et al., 2013). This predicts that both T1 or T2 turns with many abstract words may lead to longer gaps between them.

### 3.4. Surprisal

Surprisal is a measure of the amount of information a word carries about the upcoming words in a phrase. For example, the word “the” gives the listener little information about what the next word might be beyond syntactic category, while the word “helter” is almost certain to precede the word “skelter.” Various theories of processing suggest that speakers adapt their utterances to spread out the information in a sentence evenly in order to robustly transmit the signal (Piantadosi et al., 2011). In this context, the inverse of surprisal is also a measure of the “projectability” of turns (Magyari and De Ruiter, 2012) (although not necessarily of turn endings). Surprisal is conceptually the same as cloze probability (i.e., the probability of experimental participants using a word as a completion to a sentence fragment), which is used in many experiments looking at word processing (e.g., Kutas and Hillyard, 1984).

### 3.5. Syntactic complexity

Syntactically complex utterances require more processing than simpler ones. Syntactically complex sentences make greater demands on working memory (Kemper and Rash, 1988) and are harder to produce and understand (Kemper et al., 1989).

When responding to a turn, speakers must comprehend the previous turn and plan their own turn. If speakers take longer to comprehend turns with complex syntactic structures than turns with simple ones, then comprehension resources may be diverted from planning the response, making the FTO longer. At the same time, if a speaker wants to produce a complex syntactic structure, this could take more time to plan, also making the FTO longer. The prediction is that FTOs become longer as the syntactic complexity of either T1 or T2 increases.

## 4. Sequence organization

Various measures of sequence organization are discussed below.

### 4.1. Adjacency pairs

Some types of turn make a response relevant. For example, if T1 includes a question, T2 is expected to provide an answer. Answers, on the other hand, do not make the same kind of demands on the next speaker. Therefore, it is possible to identify turns that have initiating actions, like questions, and turns that have responding actions, like answers. When an initiating action, calling for a specific type of response in next turn, is followed by a relevant responding action, the turns form an adjacency pair.

The predictions about the timing of these types of turns, and whether they appear in a particular combination, are not clear. On the one hand, if initiating actions can be recognized easily, then responding actions may be produced closer to the turn end. This may be possible through the internal design of the turn (Drew, 2013; Levinson, 2013a), or through pre-ambles prior to T1 such as pre-offers (e.g., “Are you doing anything tonight?”), which set the context for initiating an offer such as an invitation. In this case, one would expect the timing of the question following a pre-sequence to be more tightly timed. Also, just as lexical frequency aids processing, so frequent adjacency pairs may be quicker to comprehend or produce. On the other hand, responding actions must “fit” with the previous turn, which may require more planning and therefore delay the response. There may also be no particular requirement in terms of timing for turns that do not form adjacency pairs.

One aspect of adjacency pairs that has been studied in terms of timing is preference (Atkinson and Heritage, 1984). Dispreferred responses, such as declinations to offers, invitations, and requests, are often delayed (Kendrick and Torreira, 2015). Delayed transitions may project the valence of the response and so allow the speaker of T1 to begin planning the third turn (the next T1) immediately (Levinson, 1983, 2013a; Clayman, 2002). For example, a delayed or hesitant response after an offer may be followed by an upgraded offer. For these reasons, although dispreferred responses themselves may be belayed, turns following dispreferred responses may have shorter FTOs.

### 4.2. Response tokens

Speakers can signal that they understand what is being said with back-channels or response tokens (Gardner, 2001). These include acknowledgement tokens (“yeah,” “mm”), continuers (“mm-hm”) and news markers (“oh,” “really?,” Heritage, 1984). While these are often produced “in the clear” they may appear in overlap without competing for the turn. Continuers, for example, are often overlapped by the prior speaker (Local, 1996; Levinson et al., 2015).

### 4.3. Laughter

Laughter has a variety of interactional uses beyond signaling joy or humor (Jefferson, 1984; Haakana, 2002; Glenn, 2003). The literature on laughter in interaction demonstrates that although laughter may occupy a turn-like slot (e.g., after a joke), laughing (or a sequence of laughter syllables) is often not treated as competing for the floor in the same way as an ordinary utterance might be, but may be superimposed on it by the speaker or be delivered in overlap by listeners (Glenn, 1989; Ford and Thompson, 1996). The lack of turn organization is indicated by the timing of laughter, which can be targeted at the content of the turn (a “recognition point”) rather than turn boundaries (Jefferson, 1974; Glenn, 1989). Therefore, laughter may often occur in overlap. Furthermore, overlapping talk is common in sequences containing laughter when humor is involved, and is not treated as problematic by the speakers. Jefferson (1974) identifies two types of laughter: a speaker may laugh after being “invited” to laugh, for instance by the previous speaker laughing, or a speaker may “volunteer” laughter unprompted. While types of laughter are difficult to code for automatically, the turns that include laughter can be identified in the Switchboard corpus. There are four possible combinations: both T1 and T2 include laughter (T1 “invites” laughter and overlap is possible); only T2 includes laughs (“volunteered” laughter, likely to be at a “recognition point” and therefore can occur in overlap); only T1 includes laughter (T1 “invites” laughter, but T2 does not respond, it is likely that T2 is an ordinary turn after a gap); neither turn includes laughter (an ordinary turn transition, therefore a gap).

## 5. Interactions between processing and sequence organization

Processing and sequence organization accounts make different predictions for some variables. For example, a faster speech rate in T1 would be predicted to lead to a longer gap due to higher processing demands in the comprehender. In contrast, some theories of turn-timing in Conversation Analysis see timing as rhythmic (Couper-Kuhlen, 1993), and would predict that faster speech rates would lead to shorter gaps.

We note that the constraints of processing and sequence organization may not be entirely disparate mechanisms. For example, Stivers et al. (2009) note that negative answers are slower. This may be because the responder is treating the answer as dispreferred (not in line with the expectation indicated by the polarity of the question), and is therefore proferring it reluctantly. But equally, it is well-known that negative responses are harder to process both in comprehension and production (Clark, 1976). In addition, frequency effects and expectability (or its converse surprisal) may apply to both processing and sequencing constraints. Certain types of turn project other types of turn. Thus, a question in T1 makes it interactionally relevant for T2 to provide an answer. Turn transitions may be shorter between these “adjacency pairs,” since adjacency pairs are more predictable and therefore aid comprehension and allow planning to begin sooner. That is, frequent, predictable structures and may aid fast transitions in the same way as frequent words do.

Speakers may overlap with an incoming turn when they wish to signal that they recognize in advance what is about to be said (so called “recognitional overlap,” Jefferson, 1986), and in tokens of agreement (Stolt, 2008). While this is an observation from the sequence organization literature, it may be measured by surprisal: words which have a large amount of information about the upcoming words allow prediction of the end of the turn.

If the timing of turn taking is the primary “ecology” to which language has to adapt (Levinson, 2006), certain processing effects may only apply after taking sequence organization factors into account. For example, planning of T2 can often begin when the pragmatic action of T1 can be recognised (Levinson, 2013a). Action ascription is often independent of syntactic structure, instead being dependent largely on sequential context (Gisladottir et al., 2012). An additional overlap between processing and conversational organization is that the latter makes systematic provision for processing problems. Thus, English makes provision for signaling a small processing hitch (uh) vs. a larger one (um) (Clark and Fox Tree, 2002, see application to the Switchboard corpus in a post by Liberman (2014), http://languagelog.ldc.upenn.edu/nll/?p=14991). Consequently, there may be an asymmetry in the predictions for the syntactic complexity of A's turn and B's turn. While T2 has no way of influencing the relationship between syntactic complexity and when T1 ends (apart from other-initiated repair), there is the option of “buffering” planning at the beginning of T2. Speakers often use turn-preserving placeholders, or hesitation markers, such as “uh” and “um” at the start of their turns to minimize the gap between turns. They may use this extra time to plan their response. This asymmetry in the options for T2 predicts that the syntactic complexity of T2 would only be correlated with the FTO when excluding initial parts of T2 that were simply turn-preserving placeholders.

In summary, the timing of turn taking may be heavily context dependent. In this case, we would not expect linear effects of processing measures over the whole data, nor simple categorical effects of sequence organization across the board. Instead, we would expect some relationships to be evident only in certain conditions. Typical regression approaches to statistical modeling are not effective at exploring this kind of data. Because of this we use a random forests framework, which can discover context-dependent relationships.

## 6. Materials and methods

Conversations were taken from the Switchboard corpus (Godfrey et al., 1992), a large corpus of telephone conversations recorded in the United States of America in the 1990s. Participants who did not know each other were connected by an automatic switchboard and were assigned a topic of conversation, which was automatically recorded. The corpus has been annotated on different levels over the years since its first release. In this study we use several layers of annotations as compiled in the NXT-Switchboard Corpus (Calhoun et al., 2010).These include segmentation of phonetic segments and words in time, which can be used to estimate the duration of turns at talk and the floor transfer between turns. Due to a flaw in the original data collection, the timing of part of the corpus is unreliable (see Calhoun et al., 2010). For this reason, recordings with unreliable timings were discarded in our study. Utterances have been hand-annotated for dialogue acts, such as yes/no questions or backchannels (Jurafsky et al., 1997). Words are annotated for parts of speech and organized into syntactic trees (Marcus et al., 1999). There is also meta-data on the speakers such as age, sex and location in the USA. Obviously, visual cues are not present in this dataset.

We processed the Switchboard files using specifically designed software (Lubbers and Torreira, 2014). This extracted the FTO between turns (Section 6.1). We categorized the dialog acts of each turn into sequence organization categories and identified turns with laughter and dispreferred responses (Sectio 6.2). For each turn in the database, we also calculated various measures of processing, such as frequency, surprisal, and concreteness, and used the syntactic annotations from the Switchboard corpus to estimate syntactic complexity (Section 6.3).

### 6.1. Calculating floor transfer offset

The corpus provides timing segmentation of phonological words (originally segmented by Deshmukh et al., 1998). We approximated “turns” by “gluing” phonological words together if they were from the same speaker and had less than 180 ms gap between them. The floor transfer offset (FTO) or “gap” and “overlap” duration between turns from different speakers was calculated using the same method as in Heldner and Edlund (2010). Transitions involving very long gaps or overlaps were discarded from the analyses (FTOs lower than -2200 ms or above 2200 ms, less than 2% of the final data). The distribution of FTOs fits well with distributions reported in other studies (see Section 7).

FTOs were also re-calculated, ignoring T2 initial turn-preserving placeholders, so that we can report FTOs with and without initial hesitation markers. These were identified as in Strömbergsson et al. (2013), as the tokens “uh,” “um,” and “well.” An alternative coding was done with identification based on the syntactic category of the initial word being an interjection, filler or discourse marker (the category “UH” from Calhoun et al.'s coding). We calculated the FTOs from the end of T1 to the beginning of the first word in T2 which was not a turn-preserving placeholder. For this set of data, T2s that consisted of only turn-preserving placeholders were excluded.

### 6.2. Sequence organization data

The Switchboard corpus is annotated with dialog acts (Jurafsky et al., 1997). These are similar to speech acts, but include categories suited for spoken conversations such as backchannels. These dialog acts were grouped into sequence types: first pair parts, second pair parts, opening and closing sequences, backchannels, repairs or “other” (see Table 1). For each dialog act type, a set of dialog acts was identified which would make a well-formed adjacency pair. For example, a yes/no question projects a yes or no answer.

**Table 1:** **The NXT dialog act categories and how they map onto sequence organization types**.

**NXT category**	**Description**	**Expected next categories**	**Initiating**	**Responding**	**Response token**	**Valence**
decl-q	Declarative Wh-Question	answer,statement	Y			
open	Conventional-opening		Y			
open-q	Open-Question	neg,affirm,no,yes,statement,reject	Y			
or	Or-Clause	neg,affirm,no,yes,statement,reject	Y			
repeat-q	Signal-non-understanding		Y			
sum	Summarize/Reformulate		Y			
tag-q	Tag-Question	neg,affirm,no,yes,statement,reject	Y			
wh-q	Wh-Question	answer,statement,reject	Y			
yn-q	Yes-No-Question	yes,no,affirm,neg,statement	Y			
yn-decl-q	Declarative Yes-No-Question	yes,affirm,statement	Y			
acknowledge	Response Acknowledgment			Y	Y	
backchannel	Backchannel			Y	Y	
backchannel-q	Backchannel as question			Y	Y	
ans-dispref	Dispreferred answers			Y		Neg
hedge	Hedge			Y		Neg
maybe	Maybe/Accept-part			Y		Neg
neg	Negative non-no answers			Y		Neg
no	No answers			Y		Neg
reject	Reject			Y		Neg
affirm	Affirmative non-yes answers			Y		Pos
agree	Agree/Accept			Y		Pos
answer	Other answers			Y		Pos
yes	Yes answers	accept		Y		Pos
apprec	Appreciation			Y		
abandon	Abandoned or Turn-Exit					
apology	Apology	agree,downplay				
close	Conventional-closing	close				
commit	Offers, Options, and Commits					
completion	Collaborative Completion					
directive	Action-directive					
downplay	Downplayer					
excluded	Excluded - bad segmentation					
hold	Hold before response					
opinion	Statement-opinion	agree,opinion,disagree,accept				
other	Other					
third-pty	3rd-party-talk					
quote	Quotation					
repeat	Repeat-phrase	agree				
rhet-q	Rhetorical-Questions	agree				
self-talk	Self-Talk					
statement	Statement-non-opinion	statement				
thank	Thanking	downplay				
uninterp	Uninterpretable					

Laughter is coded in the Switchboard transcripts, sometimes as a separate feature, and sometimes within the orthographic transcript. Turns that included laughter were identified. Preferred and dispreferred responses were identified with similar criteria as in Kendrick and Torreira (2015). Transitions where T1 initiates a question were identified (with tags “open-q,” “tag-q,” “wh-q,” “yn-q,” “yn-decl-q,” “commit”). Within these, any T2 that included an accepting dialog act (“affirm,” “yes,” “answer”) were marked as preferred responses, while all others were marked as dispreferred responses. The frequency of every possible pair of dialog acts surrounding an FTO was extracted. Obviously, the measures above are coarse approximations of the qualitative judgments of conversation analysts. However, they are useful for getting a general picture of how the principles of sequence organization could interface with principles of processing.

### 6.3. Linking the switchboard to processing measures

The turns were linked to various measures of processing. Utterance length was measured in syllables, as included in the NXT-Switchboard corpus. We calculated speech rate using the method from Wightman et al. (1992). This calculates the departure from the expected duration, calculated from the sum of mean phone durations in the corpus.

We estimated word frequency from the Switchboard corpus itself. The count of each word for each part of speech in the transcript of the full corpus was taken (the same method as Potts, 2011, except we also automatically removed tense and number inflection from nouns and verbs in order to improve the frequency estimates). The full Switchboard corpus includes around 15 million tokens. For each turn, the mean frequency of words was calculated. Larger corpora give estimations of frequency that better predict processing measures such as lexical decision times (e.g., the Subtlex corpus estimates, Brysbaert and New, 2009), but estimates are also sensitive to genre, for which the Switchboard is by definition a good match. In any case, the source of frequency estimates did not affect the general results (see Supplementary Materials 1).

Words from each turn were lemmatized and linked with concreteness ratings from a large ratings study (Brysbaert et al., 2014), matched for part of speech. A measure of surprisal was taken from Piantadosi et al. (2011), which is based on the amount of information a word contains about the following words in the Google n-gram corpus of English. For each turn, we extracted the surprisal value for each word and calculated the mean surprisal value for the turn. In addition, we estimated the uniformity of the information density by taking the mean deviation from the expected uniform information density over words.

We estimated syntactic tree depth from the NXT-Switchboard syntactic trees. The depth of a tree is the maximum number of nodes between the root and any tip in the tree. The maximum depth of any tree in a turn was taken as the maximum depth for that turn. We also measured the number of clauses in each turn, calculated as the number of “S” sentence nodes in all trees of the turn.

Altogether 19,754 turn transitions were found for which each of the 30 predictor measures were available. These came from 348 conversations involving 231 speakers, totaling around 31 h of conversation. The vast majority of the conversations lasted between four and a half and five minutes, as specified in the instructions given to participants. Speakers produced an average of 12 FTOs per minute.

### 6.4. Random forests

This paper aims to contrast measures of processing with measures of sequence organization in the explanation of turn transitions. However, many of the considered variables are highly correlated. This can invalidate the assumptions of a typical regression approach (the estimates of individual effects are unstable and the standard errors inflate, leading to misleading comparisons between the strengths of individual predictors and an under-estimation of significance of individual effects). As reported below and in the Supplementary Materials, many of the independent variables in the Switchboard data are correlated.

One solution to this problem is to use the method of “random forests” (Breiman, 2001). This is an approach based on regression (and classification), though the analyses are not linear regressions across the whole data. Instead, a “binary decision tree” (also called classification and regression tree or recursive partitioning, Strobl et al., 2009) uses the predictor variables to split the data into sub-sets. However, the structure of a decision tree is not robust to the selection of variables or sub-sets of data. In order to overcome this problem, many trees are run with sub-sets of predictor variables (hence a random “forest”), then the findings are aggregated to determine the relative importance of different variables.

First, the concept of a decision tree is reviewed. A decision tree is a hierarchy of yes/no-questions that splits data into sub-sets. To illustrate this, consider the tree in **Figure 2**. This was generated with FTO as the dependent variable and four measures of sequence organization (whether T1 includes an initiating action, whether T2 includes a responding action, whether T1 includes laughter and whether T2 includes laughter). For clarity, only the first three levels are shown.

**Figure 1:**
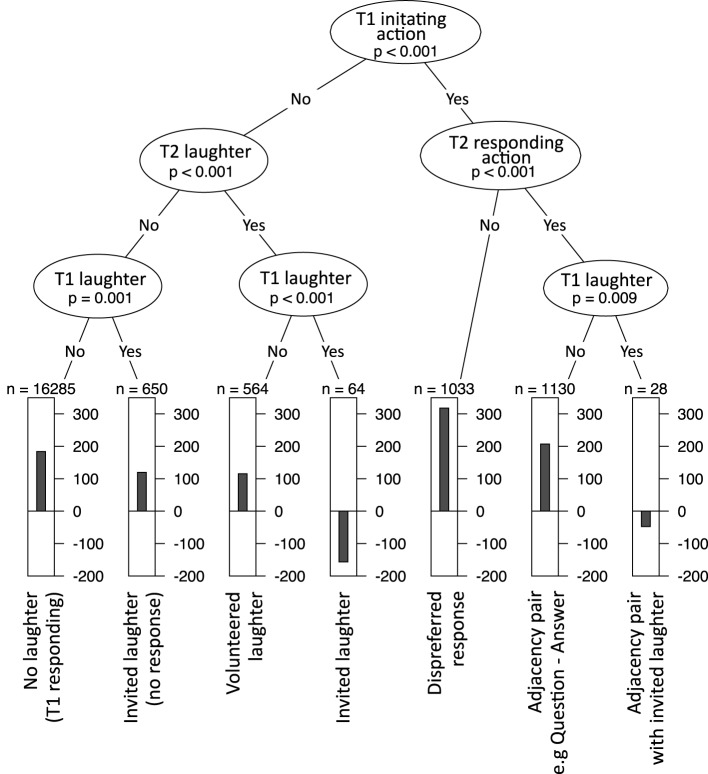
**A decision tree splitting FTO data into groups by various measures of sequence organization**.

The data is divided at each node of the tree, and the leaves of the tree show the mean FTO for that sub-set of the data in a bar chart. Above each bar chart is a number labeled *n* which represents the number of observations in that sub-set. The tree can be read like a solution to a game of “20 questions.” If you are asked to guess the value of an FTO, the decision tree aims to show you the optimal sequence of yes-no questions that will guide your guess. The tree can also be read like a set of rules that describe patterns in the data (e.g., in Figure 1, “if the turns form an adjacency pair, the FTO will be a short gap, unless there is invited laughter, in which case the FTO will be in overlap.”)

The first decision is whether T1 includes an initiating action (e.g., a question). For a given turn transition, if T1 is initiating, then we follow the right branch. The next “question” splits the data into T2s with responding actions (e.g., answers) and those without. If T2 does include a responding action, we follow the branch to the left, and are asked whether T1 included laughter. If not, then we end up at a terminal category which we might label “adjacency pair,” summarized in a bar chart. This bar chart indicates that the mean FTO is around 200 ms, based on 1130 samples (agreeing well with other studies, e.g., Stivers et al., 2009).

Every turn transition can be assigned to one of the terminal categories. For example, turn transitions where T1 is an initiating action, but T2 is not a responding action (a kind of dispreferred response) have a mean FTO of around 300 ms. This fits with work showing that dispreferred responses tend to be delayed (Kendrick and Torreira, 2015). On the other side of the tree, the questions split the data up into whether there is laughter in T1 or T2. Invited laughter, when there is laughter in T1 and T2 produces a mean FTO of around −150 ms (overlap). Again, this is in line with the literature on laughter (see above).

The algorithm that generates the tree works as follows. First, the strength of association between each predictor variable and FTO is determined by a statistical test of independence. The variable with the strongest association is chosen as the first node in the tree. The data is divided according to this variable into two sub-sets. The process repeats recursively with each sub-set until all predictor variables are statistically independent from FTO in each leaf of the tree.

The tree in Figure 1 was generated directly from data using this automatic algorithm, but exhibits many of the empirical observations in the existing literature. Variables used in decisions nearer the top of the tree have a greater influence on the outcome, so the tree would also predict that sequence type is more important than laughter.

**Figure 2:**
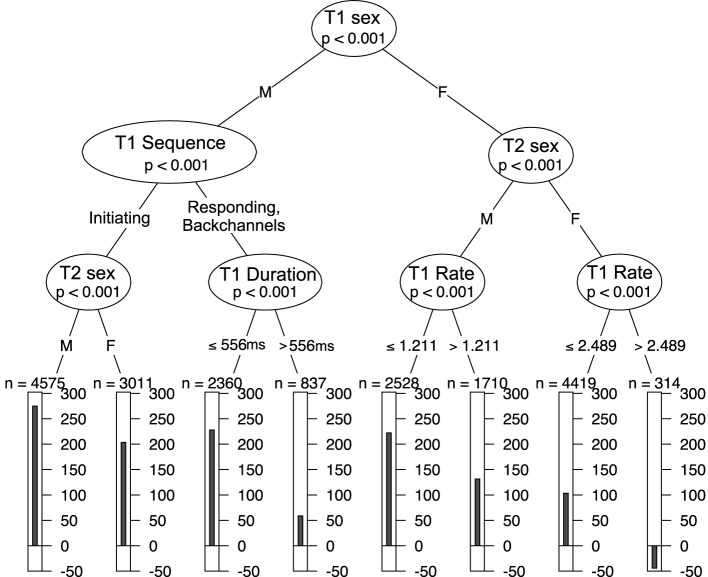
**A decision tree splitting data into gaps and overlaps by measures of sequence organization and processing**.

However, our data include continuous variables as well as categorical variables. Figure 2 shows a second tree generated with both sequence organization and processing predictor variables. The first decision is the sex of the speaker of T1. For a given turn transition, if T1 is spoken by a male, then we follow the left branch. The next “question” splits the data into T1s with initiating actions (e.g., questions) and T1s with responding actions (e.g., answers). This continues all the way down the tree, so that the leftmost bar chart shows the mean for FTOs where T1 was spoken by a male, T1 ended with an initiating action and T2 was spoken by a male. Looking at the next bar chat to the right, we see that females have lower FTOs than males when T1 includes an initiating action. For the sub-set with responding actions, we see that the duration of T1 matters, with long turns leading to shorter FTOs than short turns. This goes against the trend in the overall data for long turns to elicit longer gaps. In this way, the decision tree has separated a sub-set of data that behaves differently to the rest, and which helps explain some of the variation.

On the other side of the tree, the second decision is the sex of the speaker of T2. Comparing the leaves on the right, we see that two female talkers tend to produce lower FTOs. Speech rate of T1 is included twice on the next level—the tree cuts the continuous variable at different points for male and female T2 (variables can only be divided into two categories at any one branch, but may be further sub-divided at a later stage). This reflects the trend for males to speak faster than females. For both male and female speakers of T2, slower speech in T1 (higher T1 delta) leads to shorter FTOs. The rightmost leaf represents 314 cases of FTOs between two female speakers where T1 is speaking very slowly (high delta). In this case, the mean FTO is in overlap.

The tree in Figure 2 shows the first three levels of a full tree. A larger tree based on the full data is available in the Supplementary Materials.

One problem with decision trees is that their structures are not robust. The structure is sensitive to the selection of predictor variables and the particular sample of data (Strobl et al., 2009; Tagliamonte and Baayen, 2012). For example, the choice of the first variable may have been based on a marginal trend in the data, but may have a large effect on the subsequent choices. One way around this problem is to generate a “forest” consisting of a number of randomly generated trees. A sub-sample of the data and a selection of variables are chosen randomly for each tree. Once a large number of trees has been run, the relative importance of variables can be assessed.

We measure variable importance as the standard mean decrease in classification accuracy when a variable is permuted (see Breiman, 2001). For each tree in the forest, the prediction error (mean squared error) is calculated by comparing the true values of FTO to the values predicted by the tree. Taking the variable for which the measure is to be calculated, the assignment of each value of that variable to a case is randomly permuted and the prediction error is re-calculated. The difference between the two errors gives a measure of how influential the variable is for prediction of FTO. The difference in errors are calculated for all trees. The importance measure is then the mean of these differences normalized by the standard deviation of the differences.

The higher the importance value, the more influential the variable is in predicting the dependent variable.

For our purposes, random forests provide a way of assessing the relative importance of variables when the independent variables are highly correlated and when relationships between variables may be more complicated than simple linear patterns. Random forests have been used to look at various phenomena in linguistics (e.g., Bürki et al., 2011; Tagliamonte and Baayen, 2012; Plug and Carter, 2014; Sadat et al., 2014). Schneider (2014) analyzed the Switchboard corpus using binary decision trees and random forests to explore the distribution of hesitations in turns according to word co-occurrence frequency. Hesitations were less common between words that frequently co-occurred, supporting a “chunking” theory of language processing and production (e.g., Arnon and Snider, 2010; Bybee, 2010). However, this study did not consider the sequential organization of turns. We implement random forests using the functions *ctree* and *cforest* in the R package party (Hothorn et al., 2006a,b; Strobl et al., 2007, 2008).

Decision trees split data into subsets that can be modeled separately. That is, they try to find clusters of data that behave in similar ways. This is slightly different from linear regression which looks for linear relationships in the data as a whole. One prediction from the processing account might be that turns with low-frequency words will be responded to differently (slower) than other turns. Therefore, the tree would split the data into FTOs with high and low frequency T1s. A prediction from the sequence organization account might be that negative responses have higher FTOs, so the tree would split the data into FTOs before positive and negative T2 responses.

## 7. Results

**Figure 3:**
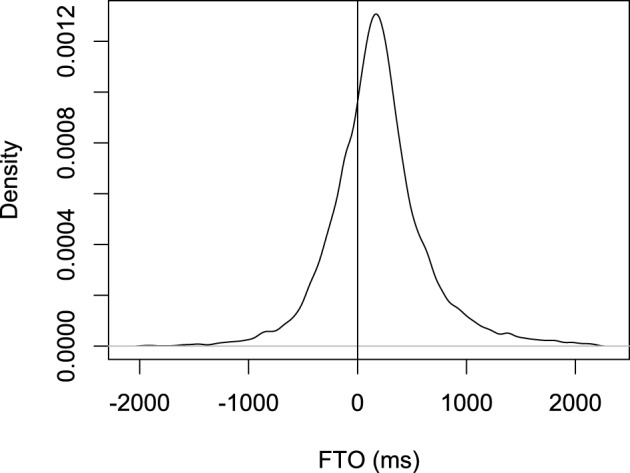
**The distribution of floor transfer offsets (the gap between two turns) for the Switchboard data**.

The distribution of FTOs is shown in Figure 3. The mean FTO was 187 ms; the median was 168 ms; the standard deviation was 448 ms; the mode (calculated by gaussian kernels with the *density* function in R set to default parameters) was 169 ms. For comparison, in our Switchboard data, the median for polar questions followed by a response was 199 ms, and (Stivers et al., 2009) found that the median FTO for polar questions followed by a response was 200 ms.

Many of the predictor variables are correlated with each other (three quarters of the variables were correlated with *p* < 0.05, see the Supplementary Materials), though there was only weak evidence for multicollinearity (maximum variable inflation factor = 3.9). The number of variables also makes the number of possible interactions very high. These two factors make simple linear regression analyses more difficult to interpret, but random forests is a robust to these concerns. Here we report various results relating to the random forests analyses.

A random forests model was run with 1000 trees and 3 variables in each tree (two runs of the model with different random stating seeds produced highly correlated variable importance measures, *r* = 0.996, *df* = 30, *p* < 0.001, suggesting that the results are robust, the results are also highly correlated when using 5 variables in each tree, see the Supplementary Materials 1). To give an impression of the fit of the model, a single tree was generated (like in the example above, but not limited to 3 levels). The predicted FTOs correlated with the actual FTOs with *r* = 0.51, meaning that the model accounts for about 30% of the variation. Another way of assessing the fit is to use the model to predict values for each FTO. When categorizing FTOs into gaps and overlaps, the model correctly categorizes 70% of cases.

In comparison, a simple linear model accounts for about 4% of the variance in FTO (see Supplementary Materials). This result is difficult to compare with linear models, since random forests work very differently (random forests are based on decision trees which divide data into sub-sets and fit each sub-set separately). Still, the difference between the two suggests that overall trends are weak, but there are more dependable patterns for certain types of transition.

**Figure 4:**
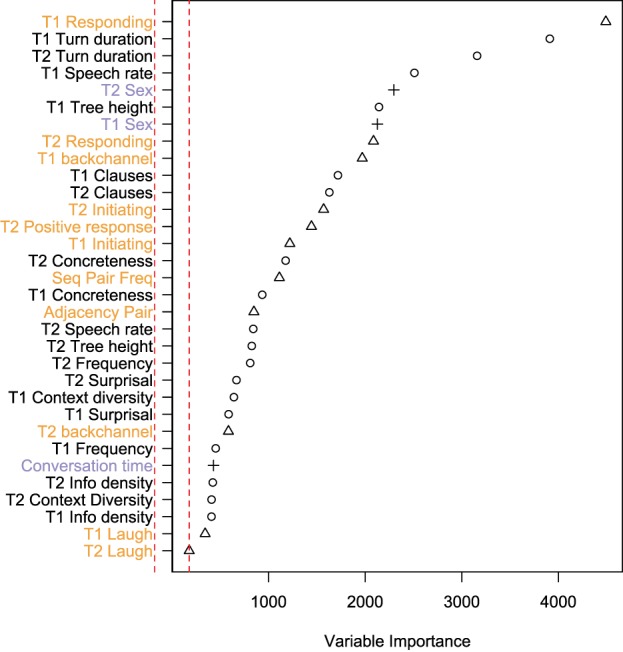
**Variable importance in a random forest analysis of floor transfer offset**. The dotted red line shows the absolute smallest value, which can be used as a baseline for spurious effects. Measures of processing appear as circles (black labels) and measures of sequence organization appear as triangles (orange labels). Other measures appear as crosses (purple labels).

Figure 4 shows the importance estimate for each variable, as calculated by the Random Forests analysis. This is an indicator of the relative importance of each variable in explaining the variation in FTO. The baseline for spurious variables is set as the absolute lowest importance measure. All variables have a positive importance value.

The top five most important variables are whether T1 includes a responding action, T1 duration, T2 duration, T1 speech rate and T1 sex. Measures of processing and sequence organization were not rated differently overall (mean importance for processing measures = 1300, mean importance for sequence organization measures = 1387, *t* = −0.21, *p* = 0.83; mean rank for processing measures = 17.8, mean rank for sequence organization measures = 16.1 *t* = 0.47, *p* = 0.64).

There was no large difference in the ranking of measures for T1 compared to measures for T2 (*t* = −0.63, *df* = 26, *p* = 0.53). For duration, speech rate and tree height the importance of the variable for T1 is greater than for T2, suggesting more weight on comprehension and planning. However, the opposite pattern holds for concreteness, frequency, and surprisal measures.

In the following sub-sections, we consider some of the most important variables, and comment on how they are related to FTO. The ranking of importance comes directly from the model results. However, the relationship with FTO is not easy to extract from the model, since a particular variable may be used to divide cases into sub-samples in very different ways. Therefore, when considering the relationship between a given variable and FTO, we explore the trends in the overall data.

### 7.1. Results for measures of processing

To give a sense of the overall trends for the processing measures, Table 2 shows the simple, linear correlation between them and FTO (more straightforward descriptive results can be found in the Supplementary Materials). Most correlations are very weak, yet, as we show below, the random forests approach does find robust patterns. This suggests that the the relationship between measures of processing and FTO is complicated: overall tendencies are weak, but more dependable patterns can be found for certain types of transition.

**Table 2:** **The Pearson correlation between processing measures and FTO for T1 and T2**.

	**T1**	**T2**
Concreteness	0.028	−0.004
Mean frequency	−0.010	0.024
Speech rate	−0.091	−0.008
Information uniformity	−0.009	−0.004
Turn duration	0.043	0.025
Surprisal	−0.003	−0.012
Number of clauses	0.026	−0.019
Syntax tree height	0.065	0.012
Contextual diversity	−0.027	−0.014

#### 7.1.1. Turn duration and rate

**Figure 5:**
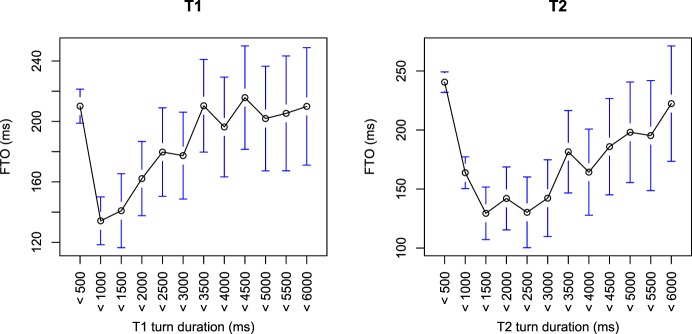
**The relationship between FTO and T1 and T2 duration**. The data is grouped into 500 ms bins. Circles represent the mean of the bin, with bars showing the 95% confidence intervals.

The variables ranked second and third most important are the durations of T1 and T2. Both T1 and T2 duration have similar relationships with FTO (see Figure 5). This relationship is non-linear: overall, longer turns occur with longer FTOs, but very short turns (less than 700 ms) are also followed by longer FTOs. The production and comprehension prediction was that longer turns would take longer to plan or comprehend, and therefore possibly start later. However, since T2 length is not linearly related to FTO, but the variable is ranked as highly important in the random forests analysis, this suggests that turn duration is being used as a proxy to distinguish different types of turn. Indeed, around three quarters of turns less than 700 ms are backchannels or agreements, while around three quarters of turns longer than 700 ms are statements, opinions, and questions. In line with this, the splits in the decision trees tend to divide data by turn duration at around 700 ms (e.g., see example decision tree in Supplementary Materials 2).

Speech rate of T1 is ranked as the 4th most important variable. On average, as T1 is spoken faster, the FTO becomes longer (this holds when excluding backchannels and short T1s). T1s spoken with rates in the fastest quartile lead to FTOs around 100 ms longer than those in the slowest quartile. The speech rate of T2 is ranked as much less important. There is no strong relationship between T2 rate and FTO.

#### 7.1.2. Syntactic complexity

T1 syntactic tree height is relatively important (ranked 6th most important out of 30), as is the number of clauses for T1 and T2 (ranked 9th and 10th). As the T1 increases in syntactic complexity, the FTO increases. Turns in the simplest quartile lead to FTOs 64 ms shorter than turns in the most complex quartile. There is no strong linear relationship between T2 syntactic complexity and FTO. The relative importance of the number of clauses in T2 may be attributed to the correlation with turn duration (*r* = 0.65, *t* = 171, *p* < 0.00001). Notice that here, as with speech rate, the processing factors only have significance in a particular sequential context, demonstrating how the two parameters, sequence organization and processing costs, are interwoven.

#### 7.1.3. Concreteness

T2 concreteness is placed in the middle of the ranking. The prediction was that turns with more concrete words will lead to lower FTOs. However, the relationship with FTO is complicated. There is no overall linear relationship. There are interactions with turn duration so that there is a positive relationship for short T2s and a negative relationship for longer T2s. This could be explained in the following way: very short turns such as backchannels tend to have very low concreteness ratings. However, some short turns, such as answers to open questions have very concrete ratings (e.g., “How many kids do you have?,” “Two”). When combined with utterance duration, then, the concreteness of T2 becomes a proxy for distinguishing response tokens (simple to project and plan) from question answers (more difficult to project and plan). Indeed, in a decision tree constructed with only T2 concreteness and T2 duration, T2 concreteness is used in a branch of the tree with short T2 turns and, within these turns, higher concreteness leads to longer average FTOs (positive relationship).

T2 concreteness seems to be more related to the absolute FTO, that is to how close the beginning of T2 is to the end of T1, ignoring whether it's a gap or overlap. There is an overall positive correlation between absolute FTO and T2 concreteness (*r* = 0.13), with the correlation being stronger as the length of T1's turn increases (for turns longer than 1000 ms, *r* = 0.23). That is the timing of turn transition is more tightly timed when T2 is less concrete (especially for longer T1s).

The relationship between T1 concreteness and FTO is more straightforward. T1s with low mean concreteness ratings are followed by short FTOs, while mid-range concreteness ratings have longer FTOs. However, T1s with high mean concreteness ratings have lower FTOs than mid-range turns.

### 7.2. Results for measures of sequence organization

#### 7.2.1. Initiating and responding actions

**Figure 6:**
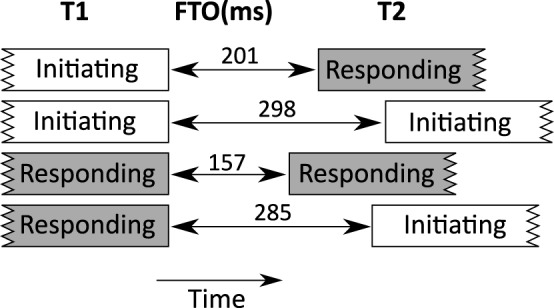
**Mean FTOs between turns with different kind of sequential actions (responding and initiating)**.

The most important factor in the whole random forests analysis is whether T1's (final) dialog act includes a responding action (e.g., an answer to a question). On average, FTOs are smaller when T1 includes a responding action (150 ms, compared to 202 ms in other cases, *post-hoc t* = 7.9, *p* < 0.00001). Whether T2 starts with a responding action, and whether T2 starts with an initiating action are also ranked as relatively important, and since they form the basis of sequence organization they are discussed together here. Figure 6 shows the mean FTOs for different combinations of T1 and T2 sequence types.

The mean FTO when T1 initiates and T2 responds (e.g., a question in T1, followed by an answer in T2) is 200.7 ms. This kind of sequence forms the basis of adjacency pairs (see Section 4.1), and agrees very well with results for polar questions from Stivers et al. (2009).

The mean FTO is longer when T1 responds and T2 initiates (284.8 ms), a floor transition involving turns which do not form an adjacency pair. For example, in the extract below, B asks a question (“lots of little funny spots, huh?”), and A gives an answer (“Oh, yeah, yeah”). This is a well-formed adjacency pair. However, if we consider the answer as T1, the next turn T2 is a different question from B. These latter turns are not part of an adjacency pair, but belong to other sequences.

Conversation 3254, 0:19

(A and B are comparing a modern adaptation of the Adams Family with the original TV series, which includes a character called Thing)


   A: Uh, there were a few things different
      than the old series, but on the, on
      the whole, it was pretty similar.
      And, a lot of fun.
   B: Lots of little funny spots, huh?
T1 A: Oh, yeah, yeah. (Responding)
   FTO = + 614 ms
T2 B: Did they have Thing, and,
      (Initiating)
   A: Oh, yes, in fact, Thing has a big,
      much bigger role than he does in the
      series.


Another possible case is that in which floor transfers occur between two initiating actions. In such cases, the mean FTO was the longest (298 ms). In our data, these often involve cases of other-initiated repair (34% of all repair initiators occur in a transition where T1 and T2 include initiating actions; 40% of turn transitions where both T1 and T2 include initiating actions involve repair). The following example is a case of other initiated repair. B asks a question (initiating action), but A does not hear or understand, and initiates a repair sequence on the previous turn. B goes on to rephrase their question, and A resumes the main question-answer sequence:

Conversation 3232, 2:13

(A and B are discussing scholarships)


   B: -- it paid most of my tuition, and,
      um, a lot of the book costs and that
      kind of thing, so.
   A: Wow, that's great.
   B: Yeah, I really,
T1 A: Was it a Pell grant? / (Initiating)
      FTO = +494 ms
T2 B: I'm sorry, what did you say?
      (Initiating)
   A: What kind of grant was it?
   B: Well, it was called a B E O G,
      a Basic Equal Opportunity Grant


In line with our results, Kendrick (2015) finds that repair initiators are delayed compared to answers to questions.

Finally, we see that the shortest average FTOs are when both T1 and T2 involve responding actions. In this case, the mean FTO is shorter (157 ms). Many of these sequences involve T1 being a backchannel. Looking closer, we also find that many are part of sequences of assessment. The example below involves a news delivery sequence (e.g., Maynard, 1997). A announces some news and B delivers a news receipt (“wow”), after which there are several elaboration turns with assessments.

Conversation 3201 3:00

(A is talking about a recycling service)


   A: But, uh, they just go around to
      each, uh, door and pick it up.
   B: Wow, that's excellent.
T1 A: Yeah. (Responding)
   FTO = +137 ms
T2 B: That's good. (Responding)


Similarly, in the next example, A responds to B's statement with an assessment in T1, and then B produces a second assessment in T2.

Conversation 3526 2:44

(A and B are talking about cheap computers sold at a local warehouse)


   B: Yeah it and when it comes on the
      manufacturing floor it's about ten
      bucks
T1 A: I'll be darned (Responding)
   FTO = +206
T2 B: Yeah (Responding)
   A: Huh well
   B: Well i watched something on TV a
      couple of months ago by uh General
      ex uh Surgeon General Koop


#### 7.2.2. Backchannels

T1 including a backchannel is ranked 9th out of 30 in terms of importance. Looking at the effect of this variable *post-hoc*, we observed that, when T1 is a backchannel, the FTO is around 38 ms lower on average than otherwise. This could occur if backchannels are regularly overlapped because they are not treated as real turns at talk (see SL & FT in Levinson et al., 2015). Indeed, the average FTO when T1 includes a backchannel is lower (with backchannel = 157 ms, without backchannel = 194 ms, *post-hoc t* = 5.43, *p* < 0.0001; 35% of T1 backchannels are overlapped by the next turn, compared to 28% of other cases). There is no big difference in means according to whether T2 is a backchannel (with backchannel = 195 ms, without backchannel = 183 ms, *post* − *hoct* = −1.9, *p* = 0.06) and this is reflected in it being ranked as relatively unimportant in the random forests results.

#### 7.2.3. Positive responses

Whether T2 provides a positive response is ranked in the middle of the distribution of importance. *Post-hoc* tests revealed that FTOs are 55 ms longer on average when T2 includes a negative response (*t* = 2.38, *df* = 1348, *p* = 0.02). This is in line with a delay for dispreferred responses, but the size of the effect is very small (the effect is weaker in a mixed effects model controlling for speaker identity and dialect), especially in comparison to the effect of T2 being a responding action vs. not.

### 7.3. Other effects

The sex of the speakers is relatively important, with each male in the conversation adding around 70 ms on average to the FTO (similar differences are obtained from a mixed effects model controlling for speaker identity and speaker dialect).

The rest of the variables have weaker importance values, but some observations are worth making. Many processing variables are not highly ranked, especially measures of information and surprisal, but also frequency, which goes against the processing predictions.

FTOs are on average lower for transitions involving laughter (mean without laughter = 192 ms, mean with laughter in T1 or T2 or both = 112 ms, *post-hoc t* = 5.4, *p* < 0.00001). FTOs are shortest when there is invited laughter: when there is laugher in both T1 and T2, the average FTO is -142 ms (overlap), as predicted by the literature on laughter in conversation. However, the laugher variables are the lowest rated variable according to the random forests analysis. This could be due to the relatively small number of cases that include laughter (about 4% of cases).

## 8. Model without turn-preserving placeholders

As discussed in Section 7, the beginnings of some turns may be turn-preserving placeholders, hesitation markers such as “um” and “uh,” that speakers use to “buffer” their response. This could obscure the demands on processing. To explore this, the same model was run, but calculating the FTO as the time from the end of T1 to the first non-turn-preserving placeholder in T2. The full results are available in the Supplementary Materials. The importance estimates in this model were weakly correlated with the main model importance estimates reported in the section above (*r* = 0.597, *df* = 30, *p* = 0.0003; rank correlation = 0.73). The prediction from processing is that the processing variables would be ranked as more important in this case, since placeholders gives responders time to plan.

The main difference in this model is that T2 turn duration has increased in importance. That is, the length of T2's turn is a better predictor of gap duration when turn-preserving placeholders are ignored. This could be evidence that speakers are “buffering” turns which require more planning. Overall, however, the processing measures do not become more important on average. Also, measures for T2 did not increase in relative importance compared to measures for T1.

Therefore, while there is some evidence that turn-preserving placeholders do buffer planning, the importance of sequential organization variables in explaining FTO cannot be easily attributed to this effect.

## 9. Discussion

This paper has examined explanations for the timing of turn taking taken either from hypotheses about cognitive processing or from those originating from sequence organization. Neither processing nor sequence organization dominated as important measures. Basic sequence organization measures such as the sequential status of turns were informative, as were measures of turn duration, speech rate, and syntactic complexity. Perhaps unexpectedly, measures of frequency and surprisal were ranked as much less important, even though they are known to affect processing and production of language in laboratory conditions. This suggests that, in real conversation, these effects often only apply in specific sequential positions, e.g., in T1 or T2, or when T1 is initiating, showing that the two kinds of account are interwoven.

One question is the suitability of the measures used. The measures of processing, for example, are not direct measures of cognitive activity, but properties of utterances that are known to correlate with processing. Having said this, the sequence organization measures are also very coarse, suggesting that this is not biasing the comparison between the two domains. Obviously, more direct on-line measurement of processing during conversation would be ideal (e.g., Holler and Kendrick, 2015). Experimental control and ecological validity are difficult to balance, but this study suggests that such an approach is warranted in the future.

In some cases, there was a difference in the predictions for processing and sequence organization accounts. For speech rate, we found that faster speech is responded to with longer FTOs. This fits with a processing account rather than a straightforward “rhythm” account, which would predict that a faster beat would lead to a faster response. Although speech rate is not the same as rhythm, and a more suitable analysis would be to code the dataset for rhythm, we still find little support for the idea that responders generally respond on the first beat, all other things being equal.

The random forests analysis explained a reasonable amount of the variance in FTOs. While it's clear that the timing of turn taking is a noisy process, the analysis suggests that there are systematic principles. The relatively good performance of the random forests analysis compared with the linear analysis also suggests that the principles are context-sensitive, rather than applying across the board. For example, certain processing effects being only evident in certain sequential contexts.

Indexical information such as the sex of each speaker was ranked as relatively important. However, these differences may not be linked inherently to sex (e.g., through processing differences), but may reflect differences in socio-cultural norms or simply individual differences.

## 10. Conclusion

We began with the observation that communication systems are imaginable, the marmoset system a putative case, in which issues of cognitive load and sequence organization play little role in influencing temporal patterns of behavior. Human communication contrasts on both dimensions, because of the formidable choice of alternatives faced by a speaker and the consequent unpredictability faced by a responder on the one hand, and on the other because sequences of ordered turns map structure onto the sheer fact that T1 is followed by T2.

By using a large coded corpus we have been able to track the importance of a set of different measures of each dimension. We conclude that the temporal patterns of dialogue cannot be accounted for by either cognitive or sequence organization factors alone. The two are interwoven with indexical factors in such a way that, for example, the sex of a speaker in a particular initiating sequence type creates an environment where cognitive load plays a particularly strong role in influencing the speed of response. This suggests that an explanation of the timing of turn taking will involve a combination of insights from both cognitive processing and sequential organization.

The ways in which factors load only in specific ecological niches make standard regression techniques inapplicable. Here the method employed, random forests, comes into its own, allowing the factor loading to be discerned in specific ecological niche formed by indexical factors, processing factors and sequence factors, as illustrated in the tree in Figure 2. The kinds of binary decision trees produced in this paper make predictions that could also be tested experimentally. There is also the possibility of using real conversational data from the Switchboard corpus as stimuli material to create a cycle of qualitative analysis and quantitative testing (e.g. Kendrick and Torreira, 2015).

This study has not exhausted the information in the Switchboard corpus. It would be possible to analyze further the different distinctions in dialog actions, such as the distinction between polar questions and content questions (initial analyses suggest that polar questions are responded to up to 100 ms sooner than content questions). There is also more indexical information such as age and dialect. Speakers were assigned topics of conversation and these may also have stimulated speakers to different degrees, which could affect average FTOs. There are also a range of phonetic information and semantic factors that could be explored. This study thus has distinct limitations. Extending the analysis to other corpora and other languages will however require large amounts of transcribed speech data, matched with processing information such as frequency and surprisal for many languages.

### 10.1. Human search and animal research

All data collected from individuals were from sources where informed consent had been provided.

### 10.2. Data sharing

Source data is available to download online (see the various references in the main text). The analysis software is also available: pympi (Lubbers and Torreira, 2014); ELAN, developed by the Max Planck Institute for Psycholinguistics (Wittenburg et al., 2006) http://tla.mpi.nl/tools/tla-tools/elan/.

## Author contributions

Extracted and prepared the data: FT; Calculated FTO: FT; analyzed the data: SR, FT; Wrote the manuscript: SR, FT, SL.

### Conflict of interest statement

The authors declare that the research was conducted in the absence of any commercial or financial relationships that could be construed as a potential conflict of interest.
