# Supplementary material for: The effects of processing and sequence organization on the timing of turn taking: a corpus study
Source: Front Psychol. 2015 May 13;6:509. doi: 10.3389/fpsyg.2015.00509 (PMC4429583; doi:10.3389/fpsyg.2015.00509)
Supplement: Supplementary file 1 [file DataSheet1.PDF]

Supplementary Material: The effects of processing and sequence organisation on the timing of turn taking: a corpus study

Seán G. Roberts [1,*], **Francisco Torreira** [1] **and Stephen C. Levinson** [1]

[1] *Language and Cognition department, Max Planck Institute for Psycholinguistics, Nijmegen, The Netherlands*

Correspondence*:
Seán Roberts
Language and Cognition department, Max Planck Institute for Psycholinguistics, Wundtlaan 1 , Nijmegen, 6525 XD, The Netherlands, sean.roberts@mpi.nl

1 DESCRIPTIVE DISTRIBUTIONS

This document describes some of the relationships between floor transfer offset (FTO) and various measures of processing and sequence organization. Caution should be exercised when interpreting the following graphs, since a simple correlation may be due to confounding factors. The main article provides a better comparison between factors.

In the graphs below, bars mark 95% confidence intervals. Table 1 shows the descriptive statistics for the continuous variables in the sample.

Variable	min	max	range	median	mean	SE.mean	std.dev
T1 Concreteness	1.19	4.96	3.77	2.45	2.28	0.005	0.67
T2 Concreteness	1.19	4.96	3.77	2.38	2.22	0.005	0.68
T1 Frequency	1.00	67584.00	67583.00	12839.49	12585.40	55.48	7798.27
T2 Frequency	1.00	67584.00	67583.00	13053.41	12793.87	58.24	8185.33
T1 Speech rate	0.00	14.12	14.12	1.05	1.25	0.00	0.69
T2 Speech rate	0.00	13.23	13.23	1.09	1.31	0.01	0.72
T1 Info density	0.00	0.21	0.21	0.01	0.01	0.0001	0.01
T2 Info density	0.00	0.21	0.21	0.01	0.01	0.0001	0.01
T1 Turn duration	60.00	40025.00	39965.00	1789.00	3170.15	26.69	3751.15
T2 Turn duration	58.00	40025.00	39967.00	1451.00	2869.70	25.44	3574.87
T1 Surprisal	1.44	15039.59	15038.15	5.88	6.75	0.79	110.88
T2 Surprisal	1.44	4106.08	4104.65	6.05	6.04	0.21	29.19
T1 Clauses	0.00	20.00	20.00	1.00	1.65	0.01	1.92
T2 Clauses	0.00	20.00	20.00	1.00	1.50	0.01	1.86
T1 Tree height	0.50	10.00	9.50	2.00	1.96	0.01	0.93
T2 Tree height	0.50	10.00	9.50	1.75	1.85	0.01	0.88
Seq Pair Freq	0.30	3.53	3.23	2.59	2.55	0.01	0.83
Duration	-1984.00	2192.00	4176.00	168.00	186.67	3.19	448.05
Conversation time	2.25	5.48	3.23	5.17	5.04	0.00	0.43

Table 1 Descriptive statistics for continuous variables in the sample.

1.1 PROCESSING MEASURES

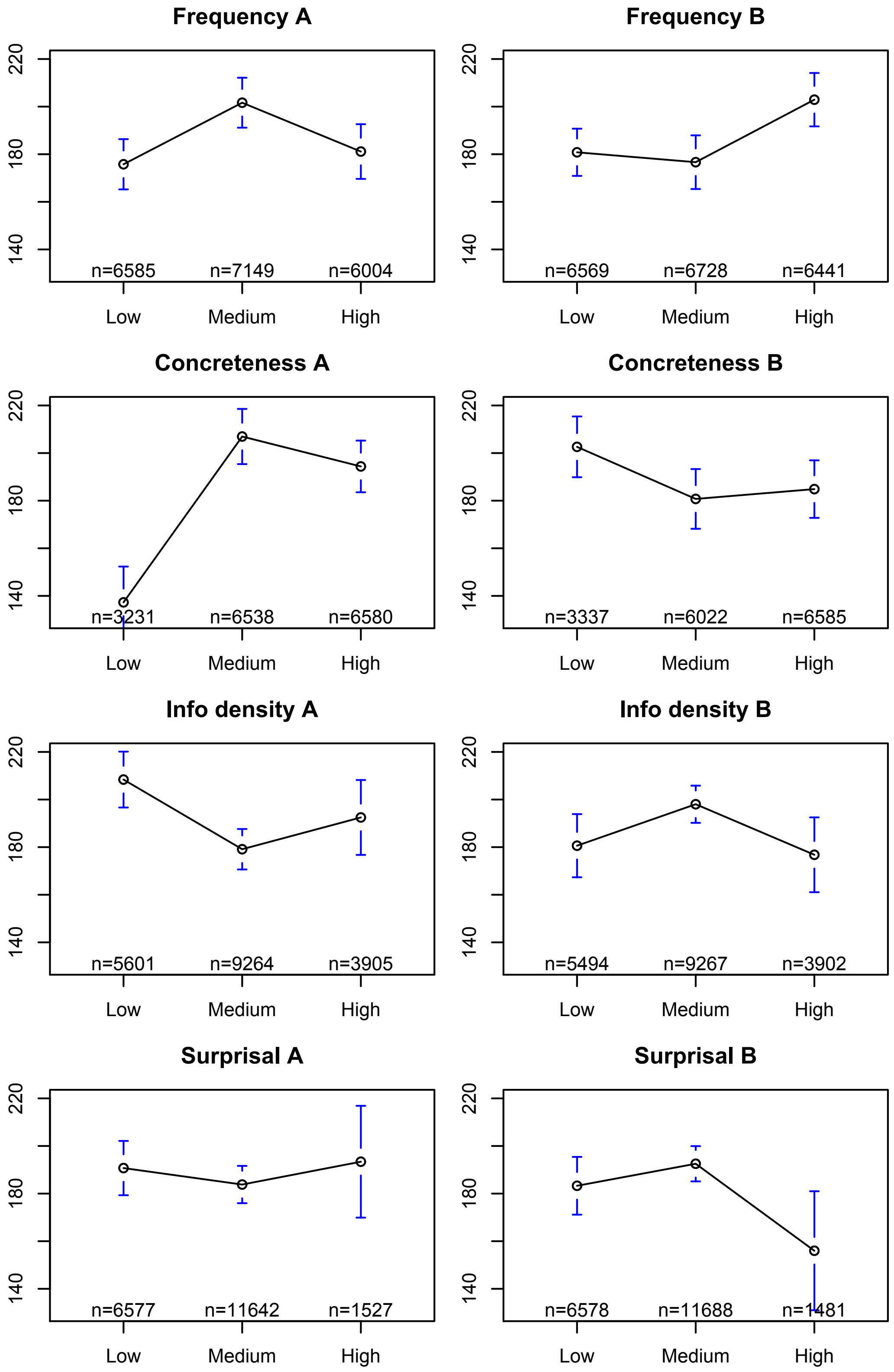

Supplementary Figure 1. How FTO varies by various processing factors. Continuous measures are split into 3 factors based on quantile. A refers to T1, B refers to T2.

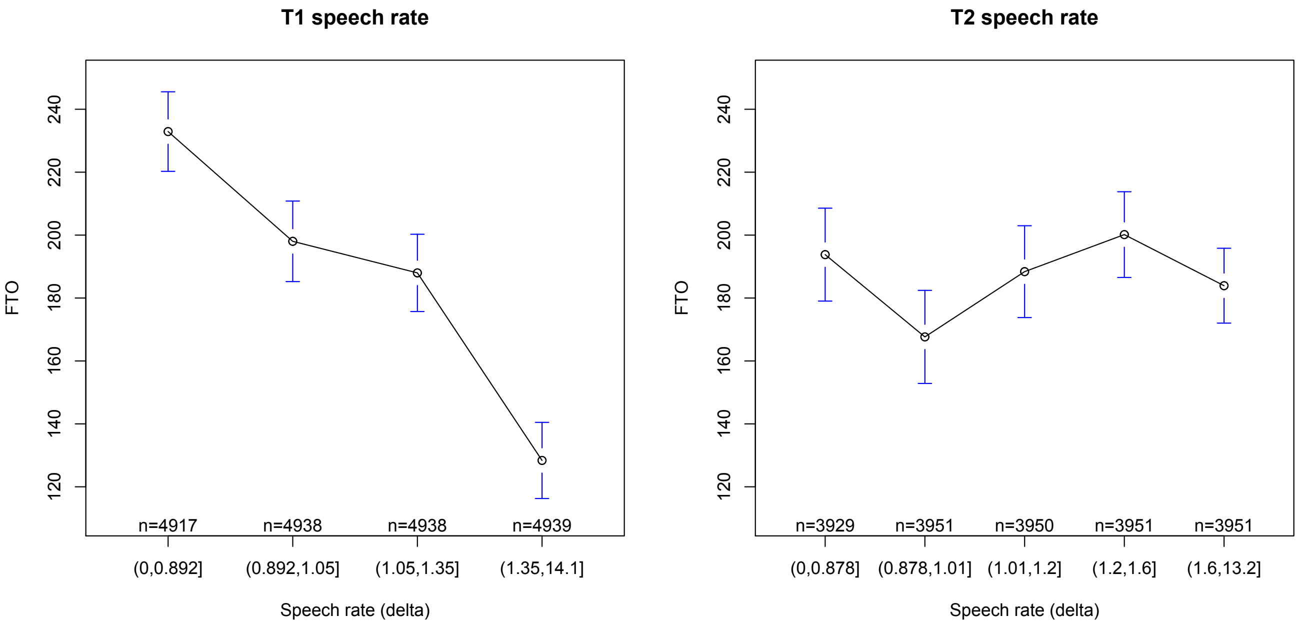

Supplementary Figure 2. How FTO varies by speech rate. The rate variable is the deviation from the expected duration. Positive values indicate slower speech, negative values indicate faster speech. The variable is cut into bins by quantile.

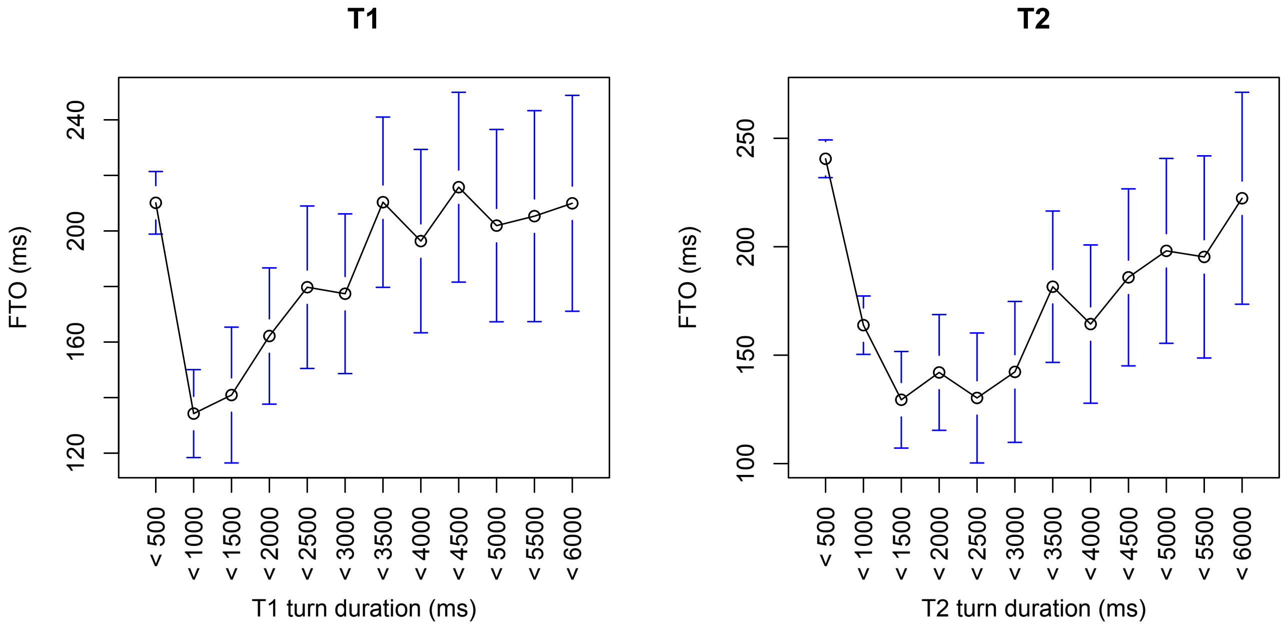

Supplementary Figure 3. The relationship between FTO and the duration of T1 (left) and T2 (right). Turns are divided into bins of 500ms. Very short turns tend to have long FTOs.
Post-hoc tests indicate that T1 and T2 duration are negatively correlated ($r = -0.16$, $t = -23.26$, $df = 19752$, $p < 0.00001$). However, this correlation is much weaker when removing backchannels ($r = -0.02$, $t = -2.2$, $df = 9961$, $p = 0.03$), suggesting that the negative correlation is being driven by long statements in T1 followed by short backchannels in T2.

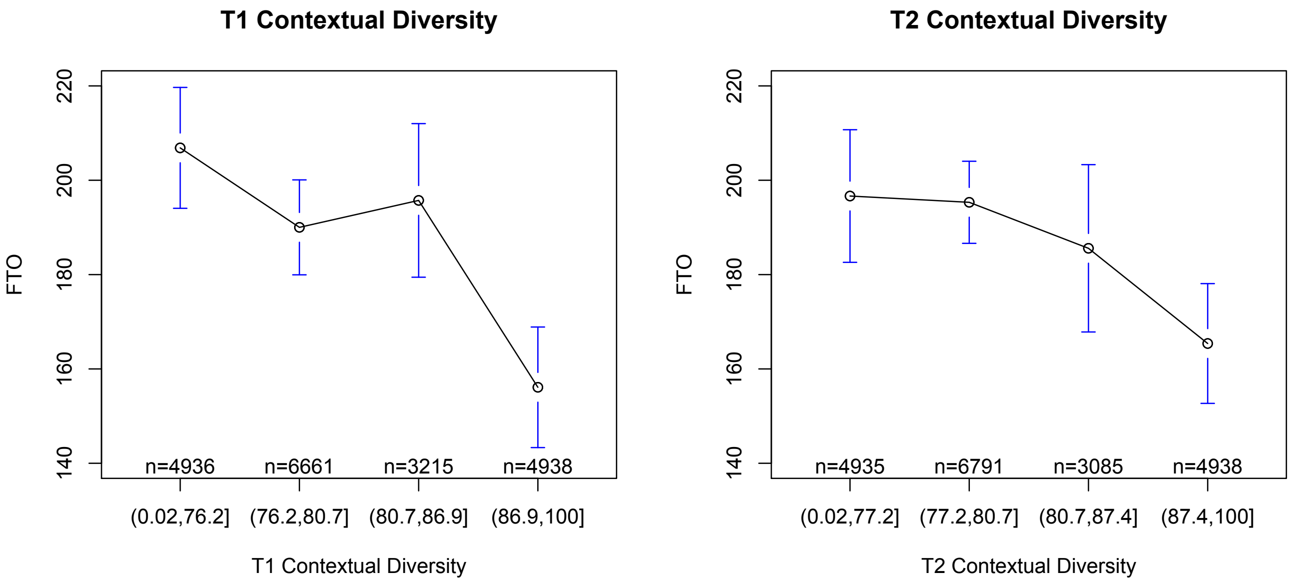

Supplementary Figure 4. The relationship between FTO and the mean contextual diversity of words in T1 and T2. Contextual diversity is measured as the proportion of films which include the given word in their subtitles, and is intended as a measure of how widely a word is used in different contexts. A higher proportion indicates a word that is used in a greater variety of contexts.

1.2 SEQUENCE ORGANISATION MEASURES

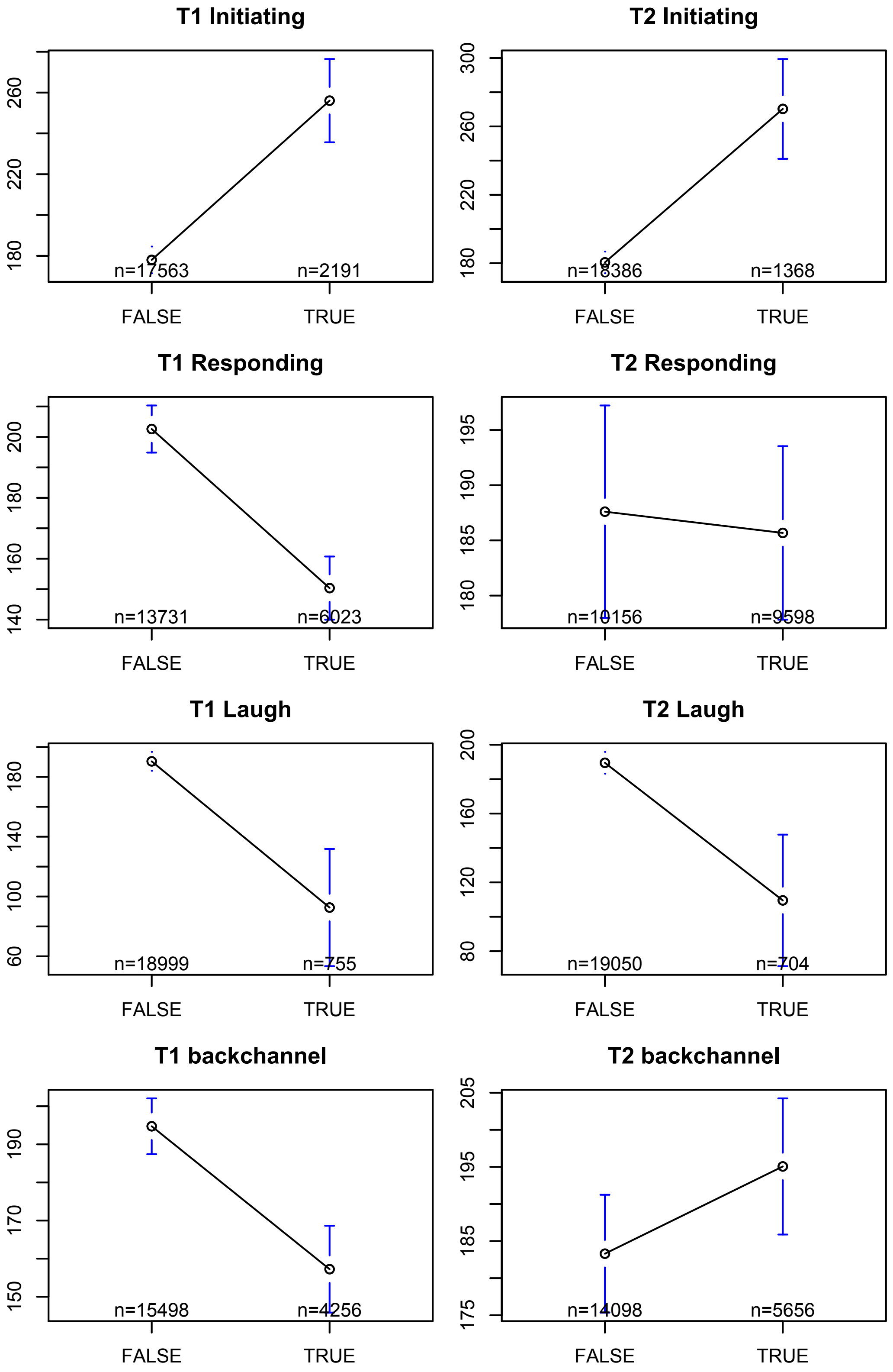

Supplementary Figure 5. How FTO varies by sequence organisation measures. Note that the scale is different for each graph.

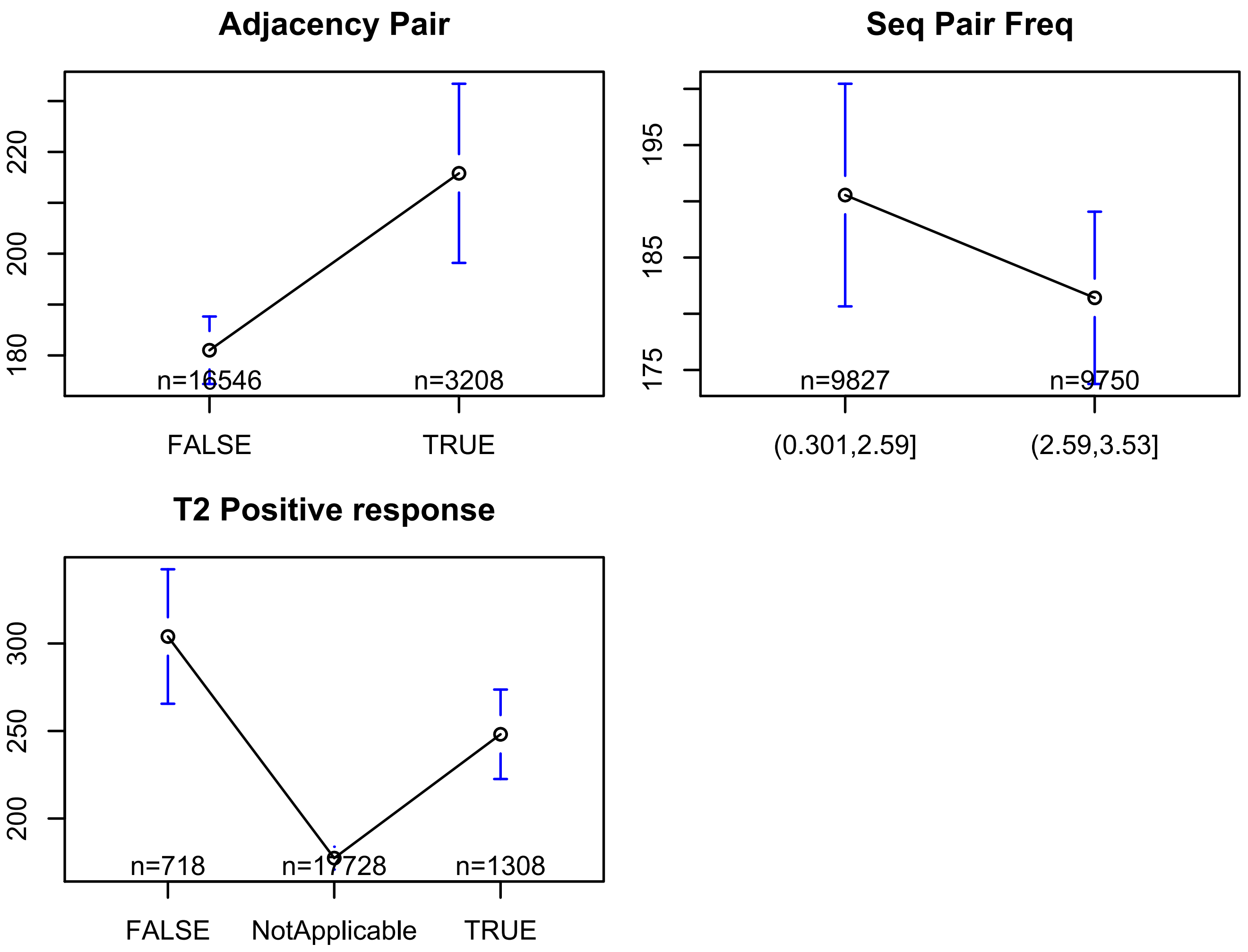

Supplementary Figure 6. How FTO varies by sequence organisation measures. Note that the scale is different for each graph.

1.3 OTHER FACTORS

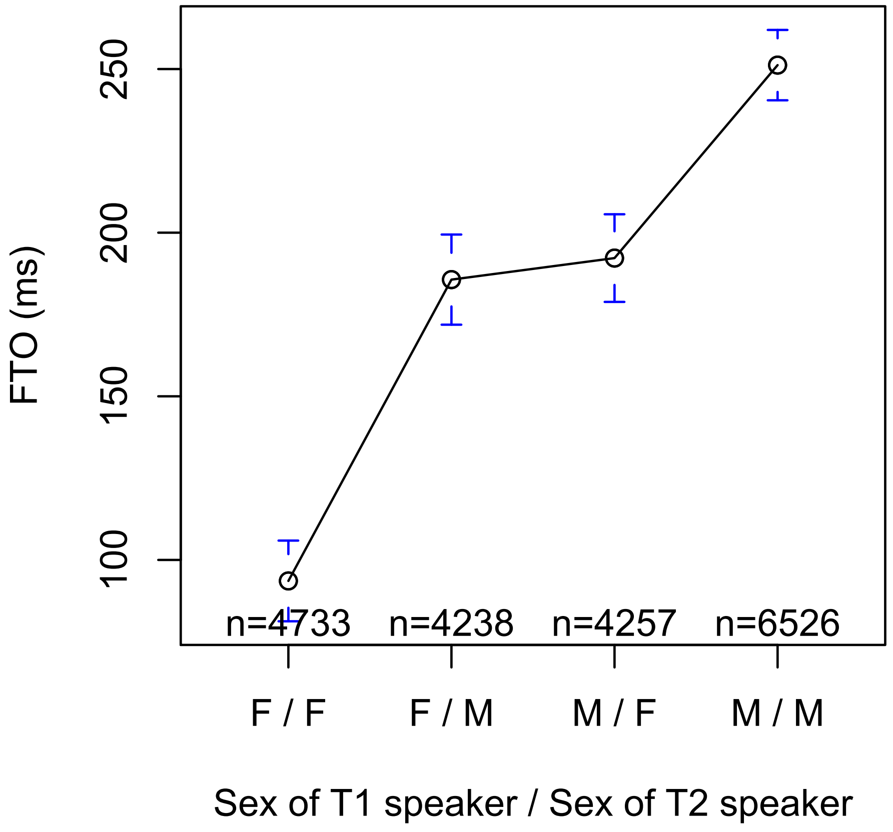

Supplementary Figure 7. How FTO varies by the sex of the speakers of T1 and T2.

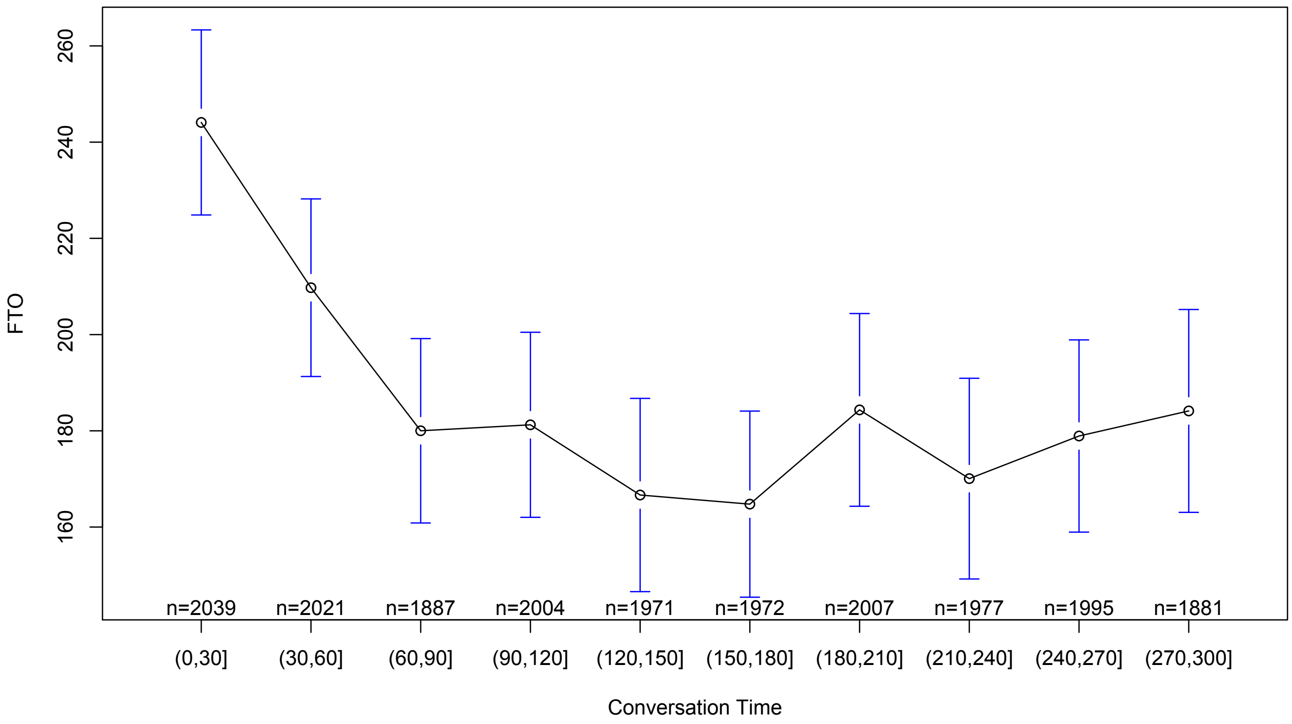

Supplementary Figure 8. How FTO varies by the point in the conversation in which it occurs. The time variable is divided into 10 bins of 30 seconds.

2 MULTICOLINEARITY

Tables 2 to 7 show the degree of association between variables. Peracon correlations, ANOVAs and χ^2 tests are used, as appropriate. 74% of variable paris are significantly related with $p < 0.05$, and 62% are significantly related with $p < 0.0001$.

Figure 9 displays these correlations graphically. It's difficult to see any clustering within domains. The relationship with speaker sex is stronger for processing variables than with sequence organisation variables (mean p-value for processing = 0.14, mean p-value for sequence organisation = 0.28, t = -2.11, df = 41, p = 0.04). We also note that sequence pair frequency is more related to the speech rate of T2 than T1. On the other hand, T1 speech rate is more related to word frequency than the speech rate of T2 (though this may be an artefact of the non-linear relationship between speech rate and mean word frequency).

The collinearity was measured using the variance inflation factor (table 8).

	T1 Concreteness	T2 Concreteness	T1 Frequency	T2 Frequency	T1 Speech rate	T2 Speech rate	T1 Info density	T2 Info density	T1 Turn FTO	T2 Turn FTO	T1 Surprisal
T1 Concreteness		-0.23 **	0.31 **	-0.1 **	-0.36 **	0.15 **	-0.01	0.03 **	0.37 **	-0.21 **	0.01
T2 Concreteness			-0.07 **	0.33 **	0.14 **	-0.33 **	0.08 **	0.01	-0.27 **	0.39 **	0
T1 Frequency				-0.02 *	0.06 **	0.01	0.08 **	0.02 *	0.12 **	-0.08 **	0
T2 Frequency					0.04 **	0.07 **	0.04 **	0.11 **	-0.12 **	0.11 **	0
T1 Speech rate						-0.07 **	0.04 **	0	-0.21 **	0.09 **	0
T2 Speech rate							-0.02 *	0.05 **	0.11 **	-0.22 **	-0.01
T1 Info density								0.02 *	-0.29 **	0.02 *	0.03 **
T2 Info density									0.01	-0.26 **	0.02 *
T1 Turn FTO										-0.16 **	0
T2 Turn FTO											0
T1 Surprisal											

Table 2. The covariance between different variables in the data. Tests include pearson correlation, anova or chi-square, as appropriate. * = significant at 0.05, ** = significant at 0.001

	T2 Surprisal	T1 Clauses	T2 Clauses	T1 Tree height	T2 Tree height	T1 Contextual diversity	T2 Contextual diversity	Adjacency Pair	T1 Initiating	T2 Responding	[illegible]
T1 Concreteness	0	0.42 **	-0.3 **	0.47 **	-0.21 **	-0.2 **	0.04 **	0.4 **	0.4 **	0.57 **	[illegible]
T2 Concreteness	0	-0.3 **	0.45 **	-0.14 **	0.48 **	0.09 **	-0.19 **	0.25 **	0.29 **	-0.7 **	[illegible]
T1 Frequency	0	0.18 **	-0.09 **	0.07 **	-0.05 **	0.1 **	0.02 *	629 **	-155.09	1584 **	[illegible]
T2 Frequency	0	-0.11 **	0.18 **	-0.06 **	0.07 **	0.05 **	0.1 **	1098 **	1141 **	-2040.76 **	[illegible]
T1 Speech rate	0	-0.28 **	0.13 **	-0.33 **	0.13 **	-0.03 **	-0.02 *	-0.3 **	-0.31 **	-0.36 **	[illegible]
T2 Speech rate	0	0.11 **	-0.3 **	0.11 **	-0.33 **	-0.04 **	-0.03 **	-0.07 **	0.01	0.4 **	[illegible]
T1 Info density	0	-0.21 **	0.03 **	-0.04 **	0.05 **	0.06 **	-0.02 *	0 *	0 **	0 **	[illegible]
T2 Info density	0	0	-0.17 **	0.03 **	-0.04 **	0.01	0.07 **	0 **	0 **	0 **	[illegible]
T1 Turn FTO	0	0.66 **	-0.22 **	0.31 **	-0.19 **	-0.12 **	0.05 **	531 **	-740.39 **	2553 **	[illegible]
T2 Turn FTO	0.01	-0.19 **	0.65 **	-0.1 **	0.36 **	0.05 **	-0.11 **	730 **	782 **	-2412.51 **	[illegible]
T1 Surprisal	0	0	0	0	0	0	0	-1.56	-1.57	1.67	[illegible]

Table 3. The covariance between different variables in the data. Tests include pearson correlation, anova or chi-square, as appropriate. * = significant at 0.05, ** = significant at 0.001

	T2 Surprisal	T1 Clauses	T2 Clauses	T1 Tree height	T2 Tree height	T1 Contextual diversity	T2 Contextual diversity	Adjacency Pair	T1 Initiating	T2 Responding	T1 Responding
T2 Surprisal		0	-0.01	0.01	-0.01	-0.01	-0.02 *	1.21 *	1.85 *	0.83 *	-0.54
T1 Clauses			-0.23 **	0.16 **	-0.23 **	-0.02 *	0.08 **	0.26 **	-0.81 **	1.49 **	-2.11 **
T2 Clauses				-0.21 **	0.23 **	0.09 **	-0.02 *	0.17 **	-0.05	-1.58 **	1.39 **
T1 Tree height					-0.12 **	-0.08 **	-0.01 *	0.79 **	1.19 **	0.54 **	-1.09 **
T2 Tree height						0.06 **	-0.09 **	0.08 **	0.1 **	-0.8 **	0.42 **
T1 Contextual diversity							0.03 **	0.26	0.42	-2.88 **	3.2 **
T2 Contextual diversity								-1.08 **	-3.36 **	3.51 **	-1.11 **
Adjacency Pair									4432 **	31.64 **	1678 **
T1 Initiating										17.73 **	1079 **
T2 Responding											4282 **
T1 Responding											

Table 4. The covariance between different variables in the data. Tests include pearson correlation, anova or chi-square, as appropriate. * = significant at 0.05, ** = significant at 0.001

	T2 Initiating	T1 Laugh	T2 Laugh	Seq Pair Freq	T2 Positive response	T1 backchannel	T2 backchannel	FTO
T1 Concreteness	0.04 *	0.23 **	0	-0.04 **	-0.43 **	-1.03 **	0.43 **	0.03 **
T2 Concreteness	0.43 **	-0.03	0.22 **	-0.24 **	-0.33 **	0.4 **	-0.82 **	0
T1 Frequency	1147 **	697 *	-403.2	0.01 *	-469.5	-2891.33 **	1663 **	-0.01
T2 Frequency	-472.67 *	154	744 *	-0.04 **	-442 **	2241 **	-2074.36 **	0.02 **
T1 Speech rate	0.05 *	0.08 *	0.07 *	-0.01	0.32 **	0.57 **	-0.26 **	-0.09 **
T2 Speech rate	-0.34 **	0.12 **	0.15 **	0.08 **	-0.07 *	-0.25 **	0.41 **	-0.01
T1 Info density	0 **	0 **	0	-0.08 **	0 **	0 *	0 **	-0.01
T2 Info density	0 **	0	0 *	-0.04 **	0 **	0 **	0	0
T1 Turn FTO	-24.82	2146 **	-520.52 **	0.14 **	234 **	-3421.53 **	2683 **	0.04 **
T2 Turn FTO	-746.03 **	-220.42	2045 **	-0.06 **	-938.91 **	1971 **	-2606.46 **	0.03 **
T1 Surprisal	-1.1	-1.23	-0.97	0.01	1.59	-0.52	3.18	0

Table 5. The covariance between different variables in the data. Tests include pearson correlation, anova or chi-square, as appropriate. * = significant at 0.05, ** = significant at 0.001

	T2 Initiating	T1 Laugh	T2 Laugh	Seq Pair Freq	T2 Positive response	T1 backchannel	T2 backchannel	FTO
T2 Surprisal	-0.7	-0.2	-0.43	0	0.07 **	-0.51	0.23	-0.01
T1 Clauses	-0.14 *	0.98 **	-0.27 **	0.14 **	0.62 **	-2.06 **	1.56 **	0.03 **
T2 Clauses	-0.69 **	-0.16 *	0.91 **	-0.03 **	-0.07	1.53 **	-1.61 **	-0.02 *
T1 Tree height	-0.05	0.14 **	-0.04	-0.06 **	-1.36 **	-1.1 **	0.34 **	0.06 **
T2 Tree height	1.16 **	-0.06	0.15 **	-0.22 **	-0.13 **	0.42 **	-0.84 **	0.01
T1 Contextual diversity	-0.02	1.25 *	0.92	-0.05 **	-1.71 **	3.74 **	-2.69 **	-0.03 **
T2 Contextual diversity	-0.21	0.99 *	1.04 *	0.04 **	2.88 **	-1.14 **	3.36 **	-0.01 *
Adjacency Pair	284 **	6.27 *	8.59 *	-0.07 **	6168 **	1050 **	1535 **	34.78 **
T1 Initiating	18.53 **	24.92 **	0.04	-0.76 **	15427 **	675 **	827 **	78.04 **
T2 Responding	1387 **	35.85 **	18.21 **	0.37 **	31.2 **	3607 **	8382 **	-1.91
T1 Responding	61.31 **	75.38 **	10.48 *	0.02	974 **	12362 **	2131 **	-52.23 **

Table 6. The covariance between different variables in the data. Tests include pearson correlation, anova or chi-square, as appropriate. * = significant at 0.05, ** = significant at 0.001

	FTO	T1 Sex	T2 Sex	Conversation time
T1 Concreteness	0.03 **	0.01	-0.04 **	-0.04 **
T2 Concreteness	0	-0.05 **	0.01	-0.04 **
T1 Frequency	-0.01	308 *	76.62	0.02 *
T2 Frequency	0.02 **	75.56	445 **	0
T1 Speech rate	-0.09 **	-0.2 **	-0.05 **	-0.01
T2 Speech rate	-0.01	-0.04 **	-0.21 **	-0.02 **
T1 Info density	-0.01	0 *	0 *	-0.05 **
T2 Info density	0	0 *	0	-0.03 **
T1 Turn FTO	0.04 **	427 **	175 *	0.04 **
T2 Turn FTO	0.03 **	106 *	333 **	-0.02 *
T1 Surprisal	0	-1.24	0.98	-0.01
T2 Surprisal	-0.01	-0.41	0.43	0.01
T1 Clauses	0.03 **	0.2 **	0.02	0.09 **
T2 Clauses	-0.02 *	-0.03	0.17 **	0.04 **
T1 Tree height	0.06 **	0.03 *	-0.01	-0.09 **
T2 Tree height	0.01	-0.03 *	0.03 *	-0.06 **
T1 Contextual diversity	-0.03 **	-1.9 **	-0.64 **	0.04 **
T2 Contextual diversity	-0.01 *	-0.64 **	-1.7 **	0.05 **
Adjacency Pair	34.78 **	0.13	7.88 *	-0.07 **
T1 Initiating	78.04 **	0.37	0.39	-0.24 **
T2 Responding	-1.91	14.79 **	0.75	0.02 *
T1 Responding	-52.23 **	25.32 **	0.41	0.04 **
T2 Initiating	89.8 **	0.73	1.38	-0.13 **
T1 Laugh	-97.79 **	59.64 **	1.98	0.04 *
T2 Laugh	-79.98 **	0.57	51.48 **	0.01
Seq Pair Freq	-0.01 *	0.01	-0.01	0.09 **
T2 Positive response	-126.61 **	2.32	0.82	0.19 **
T1 backchannel	-37.5 **	4.73 *	2.5	0.04 **
T2 backchannel	11.78	14.69 **	0.88	0.04 **
FTO		90.83 **	85.08 **	-0.05 **
T1 Sex			348 **	0.01
T2 Sex				0.01
Conversation time				

Table 7. The covariance between different variables in the data. Tests include pearson correlation, anova or chi-square, as appropriate. * = significant at 0.05, ** = significant at 0.001

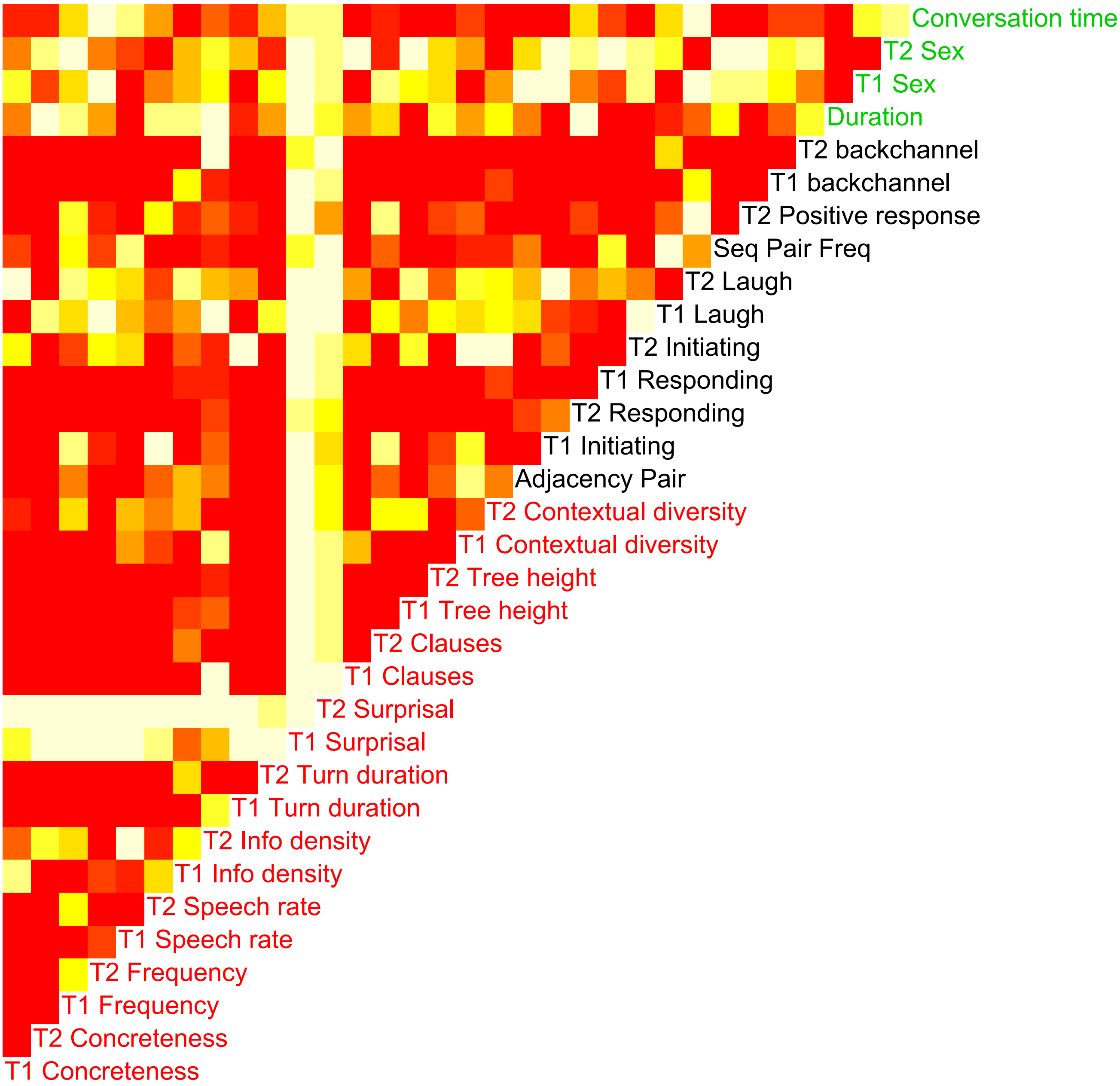

Supplementary Figure 9. Correlations between different variables. Colours represent the p-value, with warmer colours (white) indicating higher p-values and cooler colours (red) indicating lower p-values.

Supplementary Table 8. Variance inflation factors for the variables in the study.

Variable	VIF
T2 Concreteness	3.91
T2 Info density	3.81
T1 Turn duration	3.04
T1 Info density	2.47
T1 Speech rate	2.36
T2 Surprisal	2.34
T1 Surprisal	2.26
T1 Clauses	2.17
T2 Sex	2.08
Conversation time	2.04
T1 Responding	2.02
T1 Frequency	1.95
T2 Frequency	1.86
T1 Concreteness	1.8
T2 Turn duration	1.71
T2 Speech rate	1.42
T1 Initiating	1.37
T2 Responding	1.34
T1 Contextual diversity	1.27
T2 Tree height	1.23
T2 Clauses	1.22
T1 backchannel	1.14
T2 Laugh	1.14
Seq Pair Freq	1.14
FTO	1.12
T1 Laugh	1.07
T2 Initiating	1.06
T1 Sex	1.06
T1 Tree height	1.04
T2 Contextual diversity	1.03
Adjacency Pair	1.03
T2 Positive response	1
T2 backchannel	1

3 MODEL WITH 5 VARIABLES IN EACH TREE

Figure 10 shows the results of a random forest model run with 5 variables in each tree instead of 3. The model was run twice with different random starting seeds, and the variable importance measures were highly correlated across runs ($r = 0.99$, $t = 97.9$, $df = 30$, p-value < 0.0001). The model predicted FTO values that correlated with actual values with $r = 0.66$, meaning that about 44% of variance is accounted for. The model classified FTOs into gaps and overlaps correctly 73% of the time.

The importance measures of the variables for the 3-variable and the 5-variable models are highly correlated ($r = 0.99$, $t = 39.3$, $df = 30$, $p < 0.0001$).

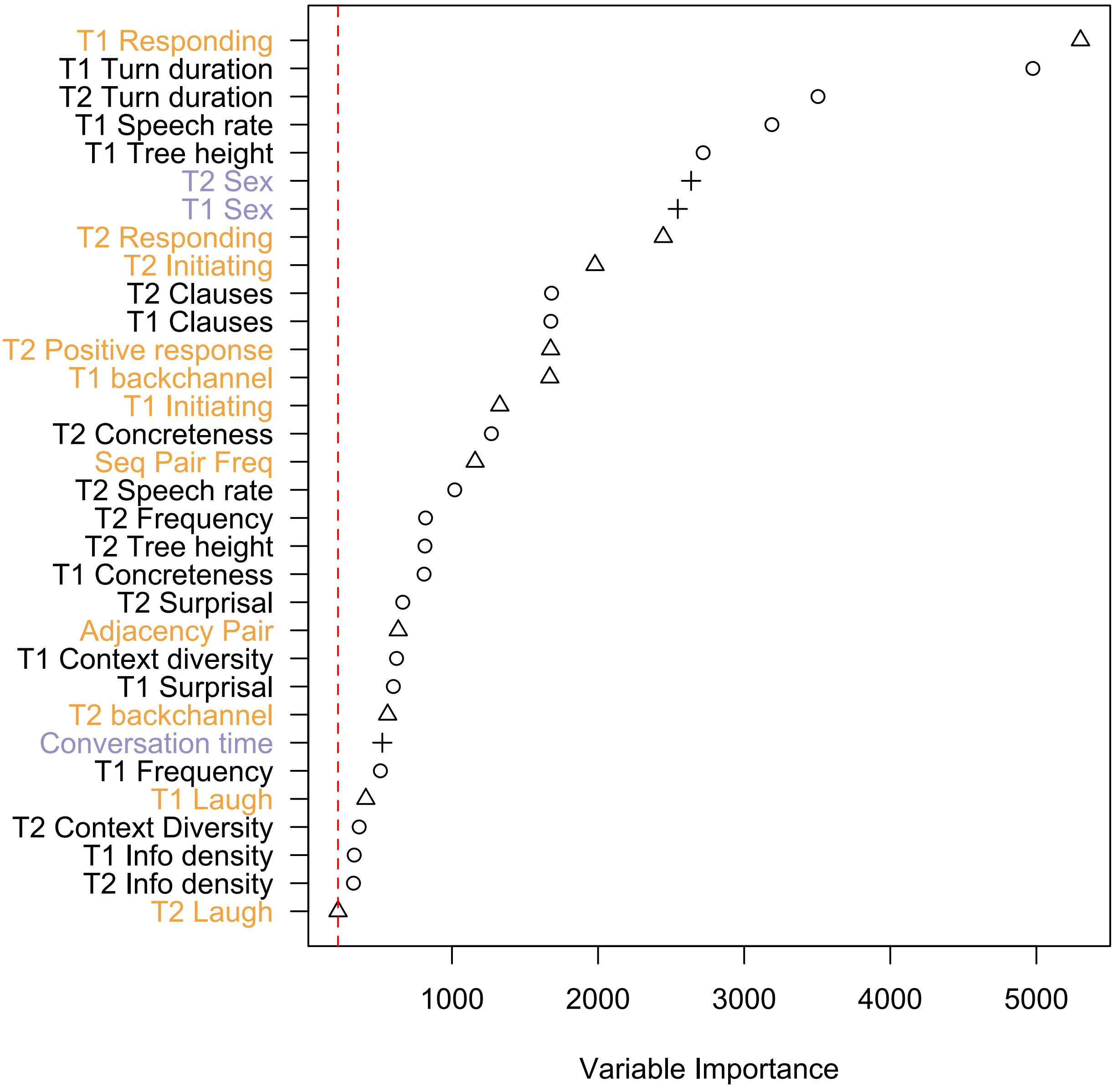

Supplementary Figure 10. Variable importance in a random forest analysis of floor transfer offset, using 5 variables per tree (instead of 3 variables as in the main analysis)

4 MODEL IGNORING TURN-PRESERVING PLACEHOLDERS

As discussed in the main paper, the beginnings of some turns may be turn-preserving placeholders, hesitation markers such as ‘um’ and ‘uh’, that speakers use to ‘buffer’ their response. This could obscure the demands on processing. To explore this, the same model was run, but calculating the FTO as the time from the end of T1 to the first non-turn-preserving placeholder in T2. The prediction from processing is that the processing variables would be ranked as more important in this case, since placeholders gives responders time to plan.

Figure 10 shows the variable importance estimates. The importance estimates in this model were moderately correlated with the main model importance estimates reported in the section above ($r = 0.597$, df=30, $p = 0.0003$), though the rank correlation is higher (Spearman rank correlation = 0.73). The main difference between this model and the main model is that in this model T2 turn duration has increased in importance. That is, the length of T2’s turn is a better predictor of gap duration when turn-preserving placeholders are ignored. This could be evidence that speakers are ‘buffering’ turns which require more planning.

However, overall, the processing measures do not become more important on average (average change in rank for processing measures = 1.8 place lower, average change in rank for sequence organisation measures = 0.05 places lower, $t = 0.77$, $p = 0.4$). Also, measures for T2 did not increase in relative importance compared to measures for T1 ($t = 0.77$, df = 21, $p = 0.45$). That is, there is little evidence that turn-preserving placeholders act as production buffers to some extent.

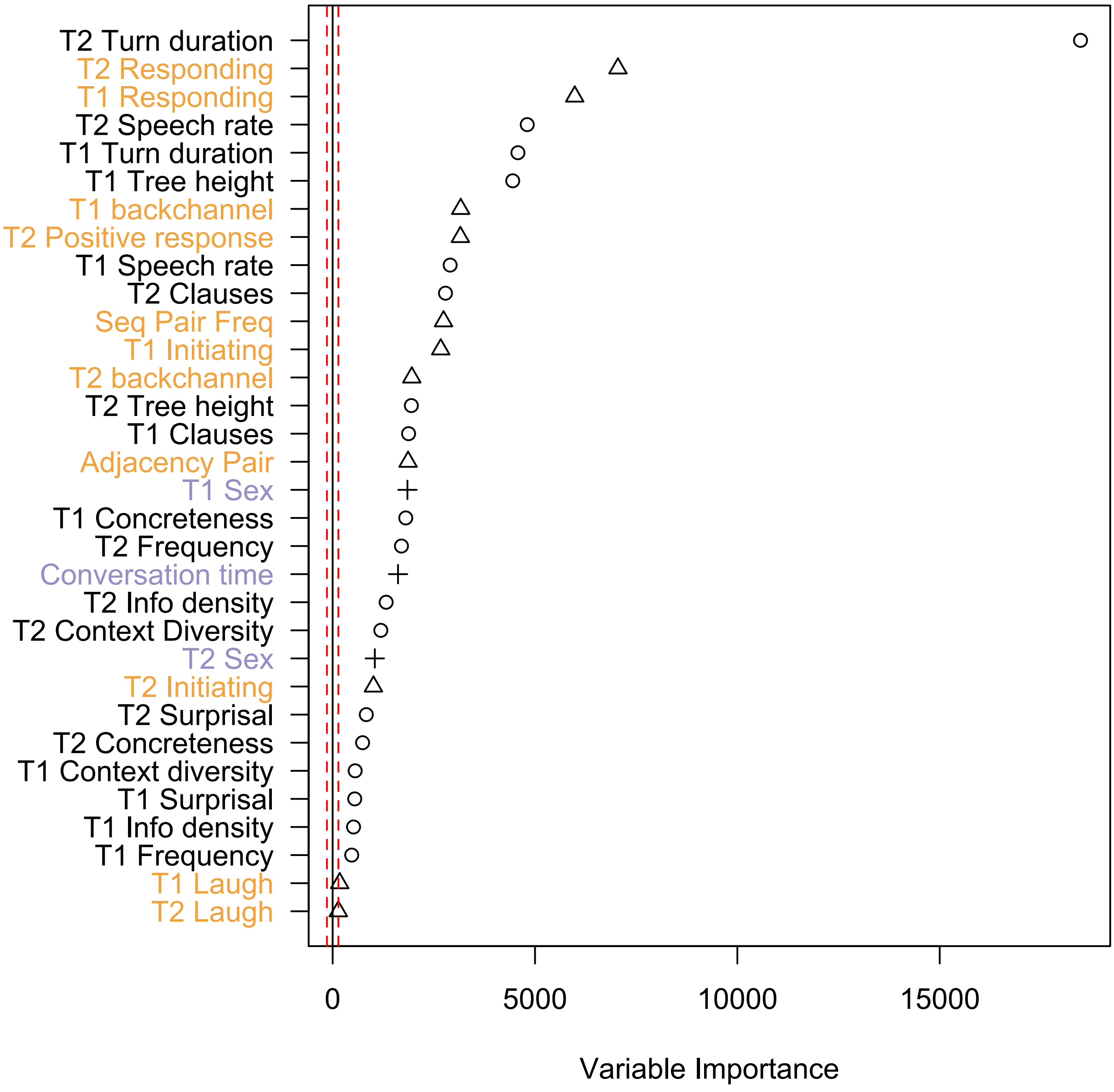

Supplementary Figure 11. Variable importance in a random forest analysis of floor transfer offset, ignoring turn-preserving placeholders. The dotted red lines show the absolute smallest value, which can be used as a baseline for spurious effects. Measures of processing appear in black (circles) and measures of sequence organization appear in orange (triangles).

5 MODEL WITH ALTERNATIVE CODING OF TURN-PRESERVING PLACEHOLDERS

A model was run with an alternative coding of turn-preserving placeholders with identification based on the syntactic category of the initial word being an interjection, filler or discourse marker (the category 'UH' from Calhoun et al.'s coding). The results are in figure 11.

Overall, the importance measures here are weakly correlated with the main model (r = 0.43, df = 30, p = 0.01; Spearman rank correlation = 0.69). The advantage of T2 turn duration is exaggerated here. Still, when comparing the main model and this alternative model, there were no clear patterns in which variables became more important, although there was a weak trend for T2 measures to improve their importance rank than T1 measures (t = -1.7, df = 18, p-value = 0.10).

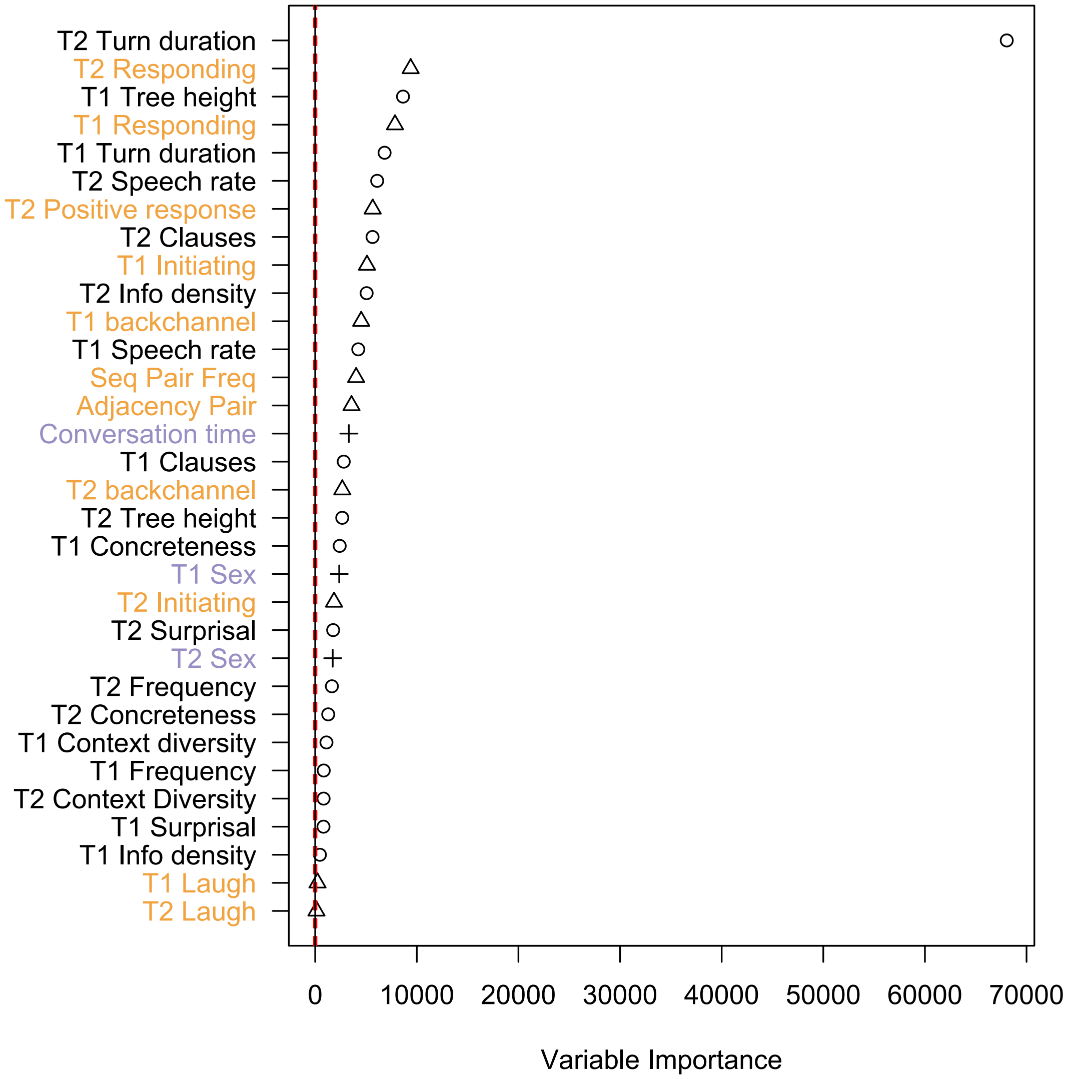

Supplementary Figure 12. Variable importance for a model run using the alternative method of identifying turn-preserving placeholders.

6 MODEL FOR ABSOLUTE FTO

A random forests analysis was run using absolute FTO (the positive amount of time from the end of T1 and the start of T2) as the dependent variable. The model accounted for about 23% of the variance in absolute FTOs. The ranking of the variables are moderately correlated with the ranking for the main (non-absolute) model (Spearman rank correlation = 0.63, $p < 0.0001$). There was no significant difference in the importance values for processing measures compared to sequence organisation measures (t = -0.0053, df = 22, $p > 0.99$). The biggest changes are for the speech rate of T1, the sex of T1 and whether T1 ends with an initiating action (less important for absolute FTO), and the contextual diversity of T2, the height of the syntactic tree for T2 and the concreteness of T2 (more important for absolute FTO). This points towards processing measures being more influential for the *accurate* timing of turn taking.

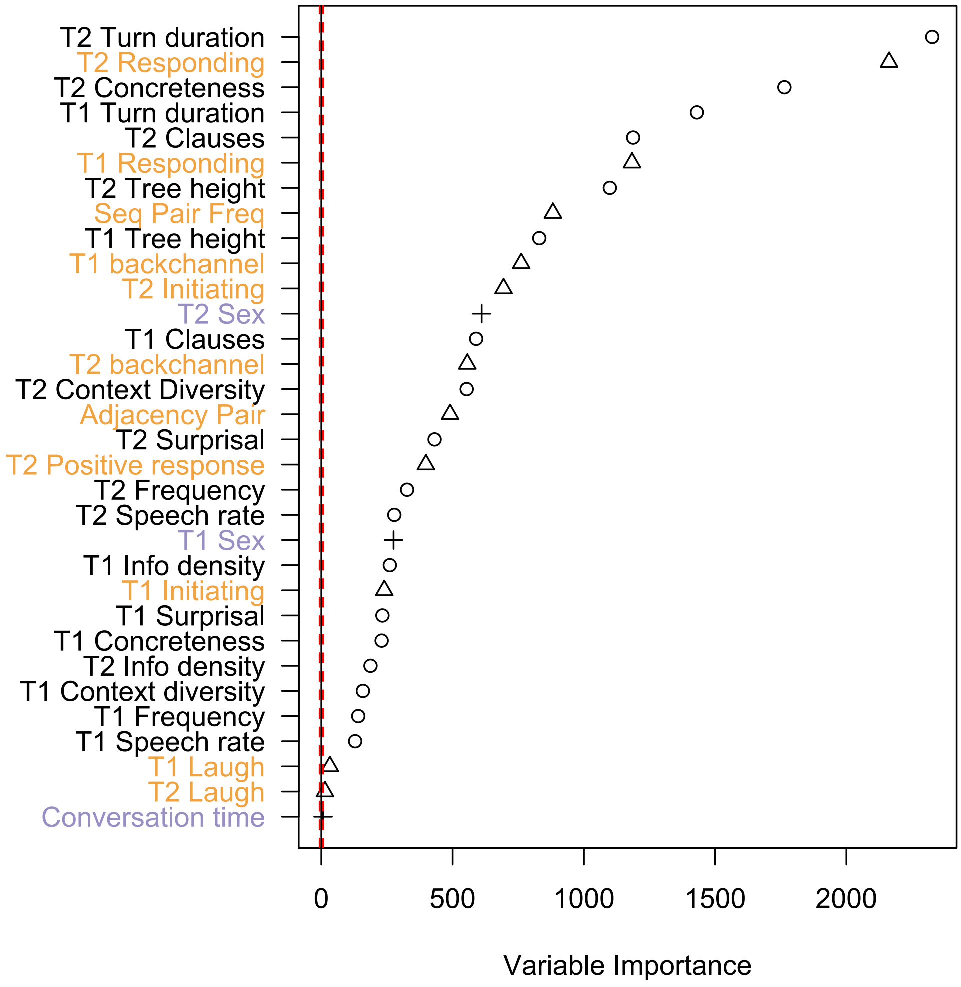

Supplementary Figure 13. Variable importance for a model predicting absolute FTO.

7 MODEL FOR POLAR QUESTIONS ONLY

849 turns were identified where T1 finished with a polar question and T1 included some responding action. A random forests analysis was done on this data. Since the sequential context was restricted, the following variables were removed from the analysis: whether turns were initiating or responding, the frequency of sequential pairs and whether the turns formed an adjacency pair.

Figure 14 shows the results. Using the minimum absolute importance value as a cut-off for spurious effects (shown as dotted red lines in figure 14), 11 variables were important: the sex of the speakers, the valence of the response, the height of T2 syntax tree, the contextual diversity of T1, information density of T2, the number of clauses in T2, T1 and T2 duration, T1 speech rate, T2 information density and T2 contextual diversity.

The model accounts for about 59% of the variance in turn transitions (correlation between FTO and predicted FTO = 0.77) and classifies 75% of cases correctly into gaps and overlaps. A linear model with the same data accounts for about 5% of the variance (not reported here).

The high ranking of the valence of the response supports the legitimacy of exploring how preference affects turn transitions (e.g. **Kendrick and Torreira**, 2015).

The most important variables reflect measures of the predictability of the question (T1 contextual diversity, T1 contextual diversity, T1 duration) and the complexity of the answer (T2 tree height, T2 clauses, T2 duration).

Figure 15 shows a decision tree built from the whole polar question data and all variables. It shows an interesting picture. It suggests that the valence of responses is important, but only for males. Looking at the raw means in figure 16, we see some support for this. However, further work would have to be done to establish whether sex is really the causal factor, or whether there are associated sociocultural factors underlying the difference.

The tree also suggests that T1 with a low mean surprisal (containing words that have little information about upcoming words) are responded to slower, while T1s that contain words that provide a lot of information are responded to faster.

However, the relationships with FTO are not always predicted according to the processing predictions. More concrete answers may be associated with longer FTOs, though this might be driven by simple ‘yes’ and ‘no’ responses being counted as abstract, so is a proxy for frequently used responses. Also, the tree suggests that in some cases (when men respond with positive, relatively abstract answers) deeper syntactic structures in T1 can cause responses in overlap (4th box plot from the left in figure 14). While the number of cases is low, this may be due to ambiguous transition relevant points in the question. Indeed, when we look at these cases, many of the T1 cases have ambiguous transition relevant points. For example, using ‘A’ and ‘B’ as possible transition points:
“Did you see it (A) the other night (B)?”
“There there enough of those (A) in the world (B), huh?”
“Okay, that’s pretty much the south end of the state (A), or the southern quarter of the state anyway (B)? ”

This results suggests that the observation from sequence organisation that the valence of the response matters for FTO timing is true, although there are also contributions from processing accounts.

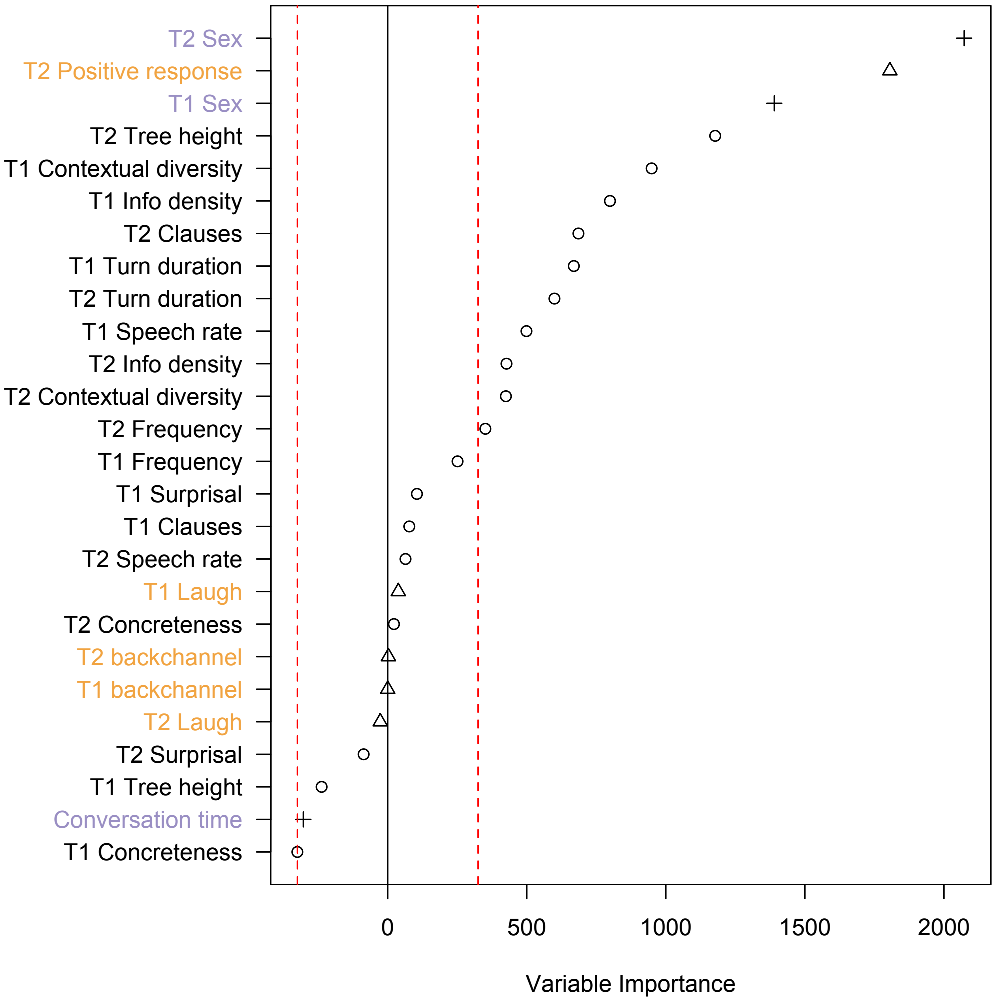

Figure 14. Variable importance for data on polar questions only. Variables with values between the dotted red lines should be considered as spurious.

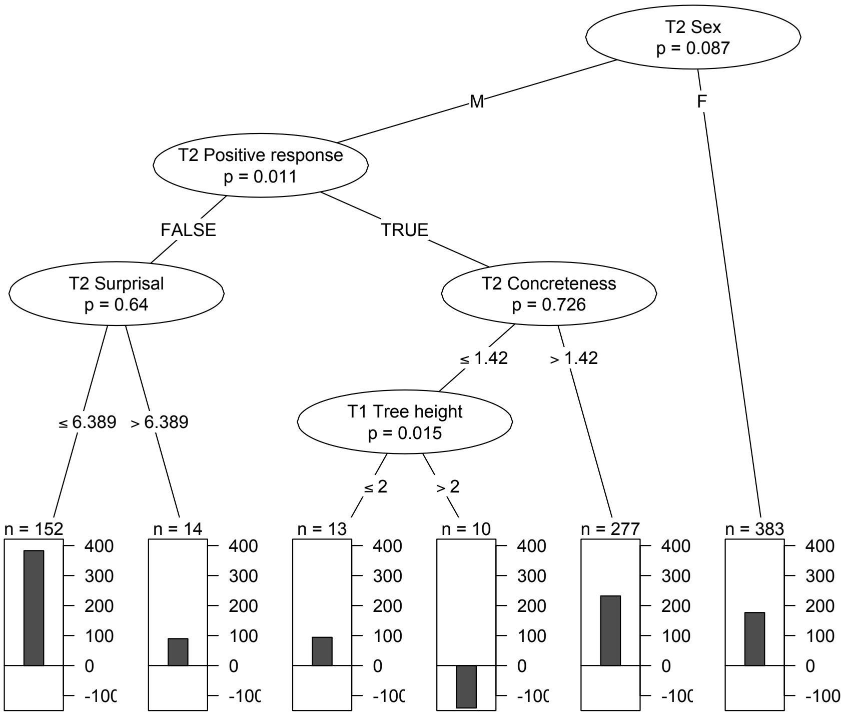

Figure 15.A decision tree for polar questions only. The criterion for the independence test p-value is relaxed to 0.2 to reveal more of the structure.

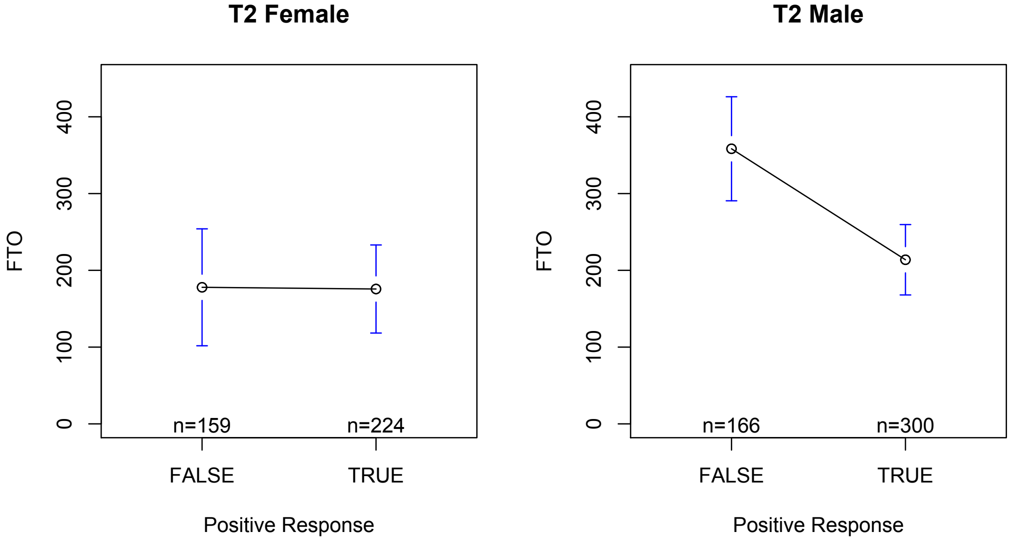

Figure 16.FTO duration by the valence of the response to polar questions, for females (left) and males (right).

8 ALTERNATIVE MEASURES OF FREQUENCY

We ran the model with frequency measures derived from the Subtlex corpus of American English **Brysbaert and New** (2009) instead of the frequency measures based on the Switchboard corpus. We extracted the frequency of the lemmatised forms of each word in each turn from the Subtlex corpus, and calcualted the mean frequency of each turn (as for the Switchboard frequency estimates). We re-ran the main random forests test with the subltex frequency measures instead of the Switchboard frequency measures. The ranking of the variables between the two types was very high (Spearman rank correlation = 0.99, Pearson correlation = 0.99). The frequency measures were ranked as slightly less important when using the Subtlex frequency measures.

9 LINEAR MODEL

A linear model was run on the same data. This model is included for comparison with the random forests model only. It is not advisable to take the linear model results at face value, since the variables are highly correlated, it fits linear relationships only and it does not account for interactions between variables.

A linear model with the same data (and all 30 independent variables) accounts for about 3.6% of the variance. Table 9 shows the results. We can rank the variables in the linear model by using the t-value associated with each variable coefficient. We can then compare the ranking of the linear model with the random forests model. The values are moderately correlated (Spearman r = 0.36, p = 0.04). The linear model ranks laughter, conversation time, frequency and backchannels as much more important, while syntactic complexity, T1 responding, sequence pair frequency, T1 speech rate and T1 duration are less important. However, a linear model is likely to be compromised by the large number of independent variables, the correlation between the independent variables and the assumption of linear relationships.

Supplementary Table 9. Resutls of a linear model run on the FTO data

	Estimate	Std. Error	t value	Pr(>|t|)
sexBM	69.679	6.476	10.760	6.29e-27
sexAM	67.088	6.469	10.371	3.9e-25
(Intercept)	561.807	65.775	8.541	1.42e-17
rateA	-43.617	5.292	-8.242	1.8e-16
B.firstInitTRUE	93.696	14.665	6.389	1.71e-10
turnDurB	0.007	0.001	5.545	2.97e-08
time.log	-39.267	7.422	-5.290	1.23e-07
laughATRUE	-77.528	16.578	-4.677	2.94e-06
dialActB.prefNotApplicable	-117.662	25.699	-4.578	4.71e-06
B.firstRespTRUE	-41.095	9.839	-4.177	2.97e-05
freqMeanB	0.002	0.000	4.081	4.51e-05
BNumS	-9.101	2.524	-3.607	0.000311
laughBTRUE	-57.381	17.123	-3.351	0.000807
backchannelBTRUE	36.418	12.073	3.016	0.00256
subtlexCDMeanA	-0.736	0.263	-2.804	0.00505
turnDurA	0.003	0.001	2.637	0.00838
dialActB.prefTRUE	-56.312	21.732	-2.591	0.00957
concMeanA	-16.429	7.141	-2.301	0.0214
A.lastRespTRUE	-27.256	13.442	-2.028	0.0426
Aheight.norm2	9.220	4.632	1.991	0.0465
backchannelATRUE	29.270	14.870	1.968	0.049
surpB.mean	-0.200	0.107	-1.860	0.063
concMeanB	-10.260	6.737	-1.523	0.128
AdjPairTRUE	15.986	11.997	1.333	0.183
Bheight.norm2	-3.727	4.642	-0.803	0.422
freqMeanA	-0.000	0.000	-0.788	0.431
A.lastInitTRUE	-17.231	21.974	-0.784	0.433
ANumS	1.515	2.506	0.605	0.545
surpA.unif2	131.307	297.648	0.441	0.659
surpA.mean	-0.010	0.028	-0.355	0.723
surpB.unif2	-82.720	280.849	-0.295	0.768
rawDiagActPairFreq	-1.187	5.342	-0.222	0.824
subtlexCDMeanB	-0.042	0.257	-0.163	0.87
rateB	0.118	5.047	0.023	0.981

REFERENCES

Brysbaert, M. and New, B. (2009), Moving beyond Kučera and Francis: A critical evaluation of current word frequency norms and the introduction of a new and improved word frequency measure for american english, *Behavior research methods*, 41, 4, 977–990

Kendrick, K. H. and Torreira, F. (2015), The timing and construction of preference: A quantitative study, *Discourse Processes*, Advance online publication. doi:10.1080/0163853X.2014.955997
