# Supplementary material for: The effects of processing and sequence organization on the timing of turn taking: a corpus study
Source: Front Psychol. 2015 May 13;6:509. doi: 10.3389/fpsyg.2015.00509 (PMC4429583; doi:10.3389/fpsyg.2015.00509)

Supplementary Figure 1:
Decision tree predicting floor transfer offset (the duration between turns) by various processing and sequence organisation measures.

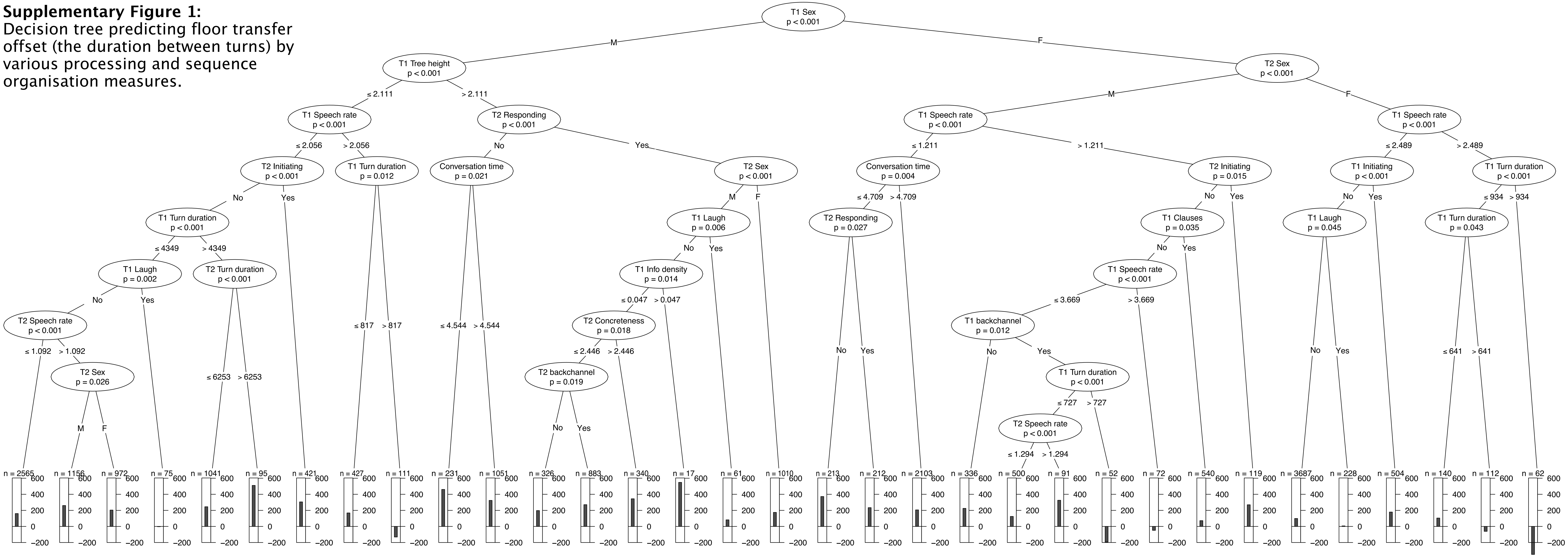

Supplement: Supplementary file 2 [file Image1.PDF]
